# Marine soundscape and fish biophony of a Mediterranean marine protected area

**DOI:** 10.7717/peerj.12551

**Published:** 2021-12-15

**Authors:** Gabriella La Manna, Marta Picciulin, Alessia Crobu, Francesco Perretti, Fabio Ronchetti, Michele Manghi, Alberto Ruiu, Giulia Ceccherelli

**Affiliations:** 1 Environmental Research and Conservation, MareTerra Onlus, Alghero, Italy; 2 Dipartimento di Chimica e Farmacia, Università degli Studi di Sassari, Sassari, Italy; 3 Area Marina Protetta Capo Caccia-Isola Piana, Alghero, Italy; 4 Department of Environmental Sciences, Informatics and Statistics, Ca’ Foscari University of Venice, Venice, Italy; 5 Nauta rcs, Milano, Italy

**Keywords:** Soundscape ecology, Biophony, Anthrophony, Boat traffic, Protected area

## Abstract

**Background:**

Marine soundscape is the aggregation of sound sources known as geophony, biophony, and anthrophony. The soundscape analysis, in terms of collection and analysis of acoustic signals, has been proposed as a tool to evaluate the specific features of ecological assemblages and to estimate their acoustic variability over space and time. This study aimed to characterise the Capo Caccia-Isola Piana Marine Protected Area (Italy, Western Mediterranean Sea) soundscape over short temporal (few days) and spatial scales (few km) and to quantify the main anthropogenic and biological components, with a focus on fish biophonies.

**Methods:**

Within the MPA, three sites were chosen each in a different protection zone (A for the integral protection, B as the partial protection, and C as the general protection). In each site, two underwater autonomous acoustic recorders were deployed in July 2020 at a depth of about 10 m on rocky bottoms. To characterise the contribution of both biophonies and anthrophonies, sea ambient noise (SAN) levels were measured as sound pressure level (SPL dB re: 1 μ Pa-rms) at eight 1/3 octave bands, centred from 125 Hz to 16 kHz, and biological and anthropogenic sounds were noted. Fish sounds were classified and counted following a catalogue of known fish sounds from the Mediterranean Sea based on the acoustic characteristic of sound types. A contemporary fish visual census had been carried out at the test sites.

**Results:**

SPL were different by site, time (day *vs*. night), and hour. SPLs bands centred at 125, 250, and 500 Hz were significantly higher in the daytime, due to the high number of boats per minute whose noise dominated the soundscapes. The loudest man-made noise was found in the A zone, followed by the B and the C zone, confirming that MPA current regulations do not provide protection from acoustic pollution. The dominant biological components of the MPA soundscape were the impulsive sounds generated by some invertebrates, snapping shrimps and fish. The vast majority of fish sounds were recorded at the MPA site characterized by the highest sound richness, abundance, and Shannon-Wiener index, coherently with the results of a fish visual census. Moreover, the acoustic monitoring detected a sound associated with a cryptic species (*Ophidion* spp.) never reported in the study area before, further demonstrating the usefulness of passive acoustic monitoring as a complementary technique to species census. This study provides baseline data to detect future changes of the marine soundscapes and some suggestions to reduce the impact of noise on marine biodiversity.

## Introduction

The marine soundscape can be defined as the aggregation of sound sources known as geophony, biophony, and anthrophony (Pijanowski et al., 2011). Geophony is among the more intense and highly variable components of the soundscape (Cato & Tavener, 1997). The sounds produced by wind, related waves, and rain have frequencies in the range between 200–2,000 Hz and 13–25 kHz, respectively (Curtis, Howe & Mercer, 1999; Ma, Nystuen & Lien, 2005; Haxel, Dziak & Matsumoto, 2013), while earthquakes and other geophysical activities dominate frequencies lower than 100 Hz (Brekhovskikh & Lysanov, 1982; Fox, Matsumoto & Lau, 2001). Biophony is produced by a wide range of taxa, from large cetaceans producing loud, long-travelling calls (Hildebrand, 2009) to small invertebrates, such as crustaceans (Schmitz, 2002). Sounds related to the presence and activities of invertebrates, as the snapping shrimp of the family Alpheidae in the frequency range between 2 and 10 kHz (Au & Banks, 1997) or the grazing activities of sea urchins in the frequencies between 850 and 2,500 Hz (Radford et al., 2008a), often dominate the soundscape of rocky reefs. Furthermore, over 800 species of fishes worldwide have been identified as vocal (Rountree et al., 2006) and the communicative role of their sounds has been widely studied (Ladich, 2004; Ladich & Winkler, 2017; Ladich, 2019). Fish sounds are known to be associated with feeding, territorial, or reproductive behavior, and the rate of sound production and acoustic repertoire change in accordance with behaviour (Amorim, 2006). The majority of sounds produced by fishes are made up of repetitive pulses with a frequency that is typically below a few kHz (Amorim, 2006; Fine & Parmentier, 2015). These acoustic features may allow for their distinction in marine soundscapes and, when the identity of the emitter is known, the passive acoustic monitoring (PAM) technique represents an effective way to map the fish presence with no impact on ecosystems. Moreover, PAM systems can be applied to record acoustic data on large spatial and temporal scales without human presence or limitations related to night hours or weather conditions (Aran Mooney et al., 2020). Many sounds with acoustic features attributable to fish species still have unknown sources. Thus, further research to associate these sounds with the emitting species is fundamental, both to advance bioacoustics (Wall et al., 2013) and to increase PAM use effectiveness.

Thus far, the assessment of fish biophony in the marine soundscape has been used for different purposes, such as to (i) describe reproductive and spawning behaviour at sea (Fine & Thorson, 2008; Locascio & Mann, 2008; Luczkovich, Pullinger & Johnson, 2008; Picciulin et al., 2013; Casaretto et al., 2014), (ii) identify essential fish habitats, temporal, and spatial distributions (Luczkovich et al., 1999; Wall et al., 2013; Di Iorio et al., 2018; Parmentier et al., 2018; Bertucci et al., 2020), (iii) track vertical fish migrations (D’Spain & Batchelor, 2006), and (iv) estimate the occurrence of cryptic fish species (Kéver et al., 2016; Picciulin et al., 2018a). The soundscape analysis, in terms of collection and analysis of acoustic signals, has also been proposed as a tool to evaluate the specific features of ecological assemblages and to estimate their acoustic variability over space and time (Sueur & Farina, 2015). In this context, acoustic diversity has been associated with ecosystem health (Bertucci et al., 2016), and fish sounds have been used as proxy for assemblage complexity (Di Iorio et al., 2018; Aran Mooney et al., 2020). For example, some studies found a relationship between taxonomic and acoustic diversity (Kaplan et al., 2015; Staaterman, 2017; Kaplan et al., 2018). Although the species emitting several different sound types have not yet been identified in the Mediterranean Sea (Ruppé et al., 2015; Carriço et al., 2019), Desiderà et al. (2019) highlighted that fish sound type diversity itself provides, a measure of taxonomic diversity of an ecological community. Furthermore, Di Iorio et al. (2018) proposed a type of sound, referred to as “kwa”, dominating the soundscape of Mediterranean *Posidonia oceanica* meadows, as an environmental proxy for habitat monitoring, despite that its attribution to *Scorpaena* spp. has only been lately established (Bolgan et al., 2019). The detection and characterization of marine sounds are still fundamental to describe the acoustic biodiversity of any habitat (Lindseth & Lobel, 2018), even if different acoustic indices have been developed with the aim to summarize the information included in the soundscapes and to correlate them with local biodiversity (Sueur et al., 2014; Bohnenstiehl et al., 2018; Pieretti & Danovaro, 2020). These indices have been applied to temperate and tropical marine areas (*i.e*., Staaterman et al., 2014; Harris, Shears & Radford, 2016; Bertucci et al., 2016; Pieretti et al., 2017; Ceraulo et al., 2018; Elise et al., 2019), with contrasting results (Aran Mooney et al., 2020).

Anthrophony is a relevant component of marine soundscapes and comprises sounds produced by ship and boat traffic, sonar, pile driving, explosions, seismic exploration, dredging, and port construction. The increase in ocean noise over the past few decades (McDonald et al., 2008) has led to the regulation of underwater acoustic pollution in different international legislations (US National Environment Policy Act, the Marine Strategy Framework Directive). In particular, the European MSFD (2008/56/EC) introduced noise to the good environmental descriptor (GES) 11. Namely, the descriptor 11.2 refers to the “continuous low frequency sound” and suggests the monitoring of the ambient noise level at the 1/3 octave bands centred at 63 and 125 Hz. In coastal areas, boat traffic is among the main source of noise (Graham & Cooke, 2008) in the frequencies between 100 and 1,000 Hz (Erbe, 2002, 2012). These frequencies mostly overlap with those of fish sounds and fall within the auditory capability of many fish and marine mammals (Hildebrand, 2009), affecting marine animal physiology, communication, behaviour, and energetics (reviewed by Rako-Gospić & Picciulin, 2019).

Marine Protected Areas (MPA) are commonly tourism hot spots due to the high quality of the environment, the scenic landscape, high habitat diversity, and species richness. At the same time MPAs are considered the most effective tool for reducing anthropogenic disturbances to marine resources and habitat degradation, and for maintaining the good conservation status of fish and mammals. Therefore, it seems impelling to measure how human activities, such as boat traffic, within the MPAs can affect the natural environment (La Manna, Manghi & Sarà, 2014), although understanding the impacts of noise pollution on marine ecosystems is still limited by the paucity of available data on marine soundscapes. Even if the MPAs are sites where boat traffic and related noise could be significantly managed and regulated (Codarin et al., 2009), few protected areas perform monitoring actions to properly address the management of boat traffic and acoustic pollution (Haren, 2007).

The Capo Caccia–Isola Piana MPA (hereafter CCIP MPA) is located along the west coast of Sardinia, in the centre of the western Mediterranean Sea. It includes a Site of Community Importance (SCI IT01B10042) and, since 2009, was also inserted in the SPAMI list because of its particular relevance related to the presence of ecosystems and habitats of endangered species specific to the Basin and its elevated scientific, aesthetic, cultural, and educational values. Recent studies in the area highlighted the presence of 59 fish taxa (belonging to 20 families) including same soniferous species of conservation value, such as the brown meagre (*Sciaena umbra*) and grouper (*Epinephelus marginatus*) (Manghi et al., 2020; Grech et al., 2020; La Manna et al., 2021). This MPA is also part of the home range of a resident population of common bottlenose dolphin (*Tursiops truncatus;* La Manna et al., 2020b; Frau et al., 2021).

The identification and measurement of the natural and anthropogenic sources that form the soundscape are essential for assessing the impact of anthropogenic disturbances on marine habitats and species, especially in marine protected areas. Recent studies in the CCIP MPA have highlighted the impact of boat noise on some marine protected species, such as *Tursiops truncatus* (La Manna et al., 2019, 2020a) and *Sciaena umbra* (La Manna et al., 2016). For this reason, underwater noise and boat traffic monitoring have been included among the research and monitoring objectives of SCI IT01B010042 Management Plan. Thus, the main aims of this study were to characterise the CCIP MPA soundscape over short temporal and spatial scales, and to quantify the main anthropogenic and biological components, with a focus on fish biophony. The results implement information to enhance the local conservation of ecosystems and provide baseline data to detect future changes in the marine soundscape.

## Materials & Methods

### Schematic overview of experimental program

**Figure 1:**
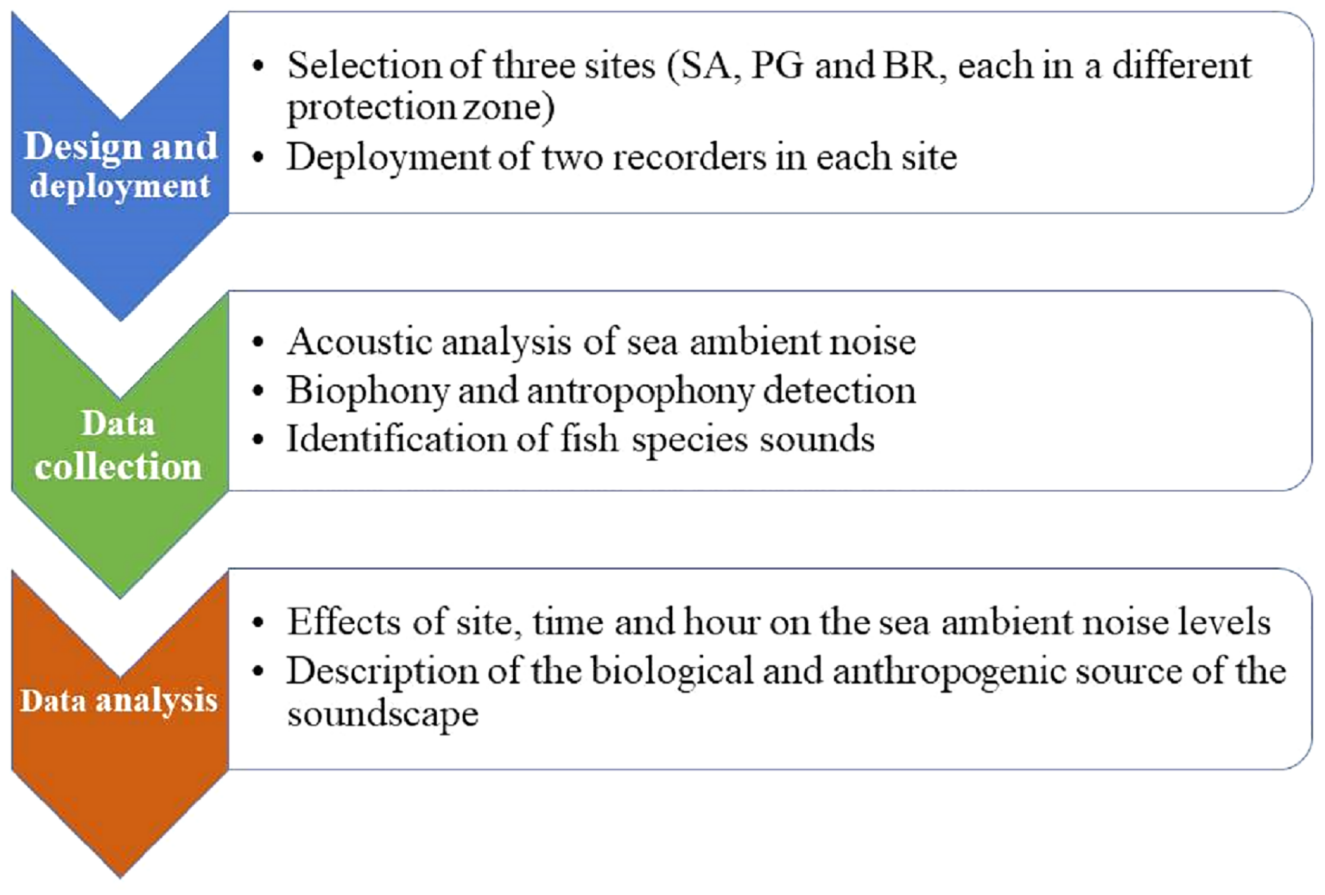
Flow chart. Schematic overview of experimental program.

The experimental program consisted of a few simple steps: the selection of sites, deployment of recorders, acoustics, and then statistical analysis. One site for each of the MPA zones (integral, partial, and general protection) was chosen. At each site, two acoustic recorders were deployed on a rocky bottom with some seagrass patches. Based on the sound frequencies, sea ambient noise was characterized by detecting the contribution of biophony and anthrophony. Fish sounds were classified and counted following a catalogue of the known soniferous species from the Mediterranean Sea, based on the acoustic characteristic of the sound type. The analysis of data tested for the effect of site, time (day *vs* night), and hour (24 levels) (Fig. 1).

### Study area and data collection

**Figure 2:**
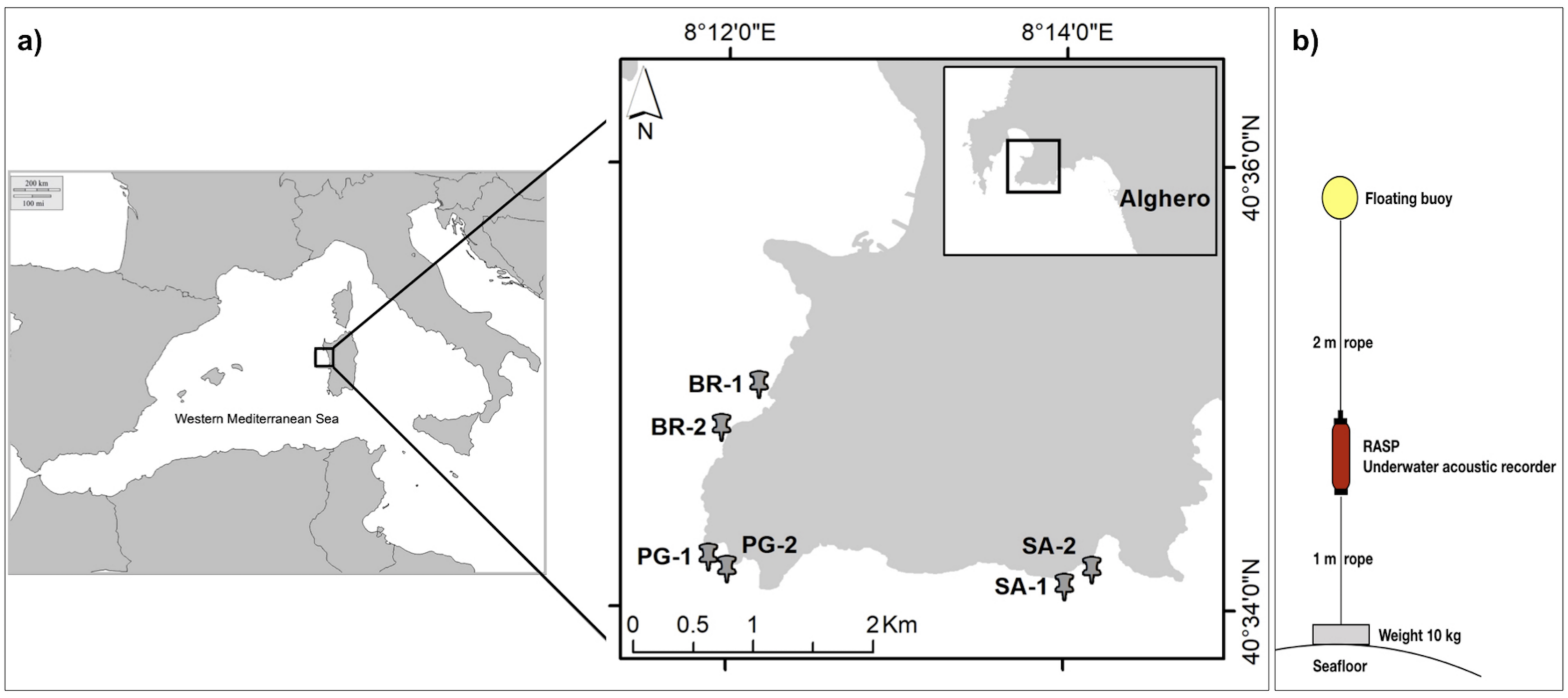
Study area and acoustic recorder setup. (A) Study area. Sampling sites (BR, Bramassa; PG, Punta Giglio; SA, Sant’Antonio) and areas (1 and 2); (B) Autonomous Underwater Acoustic Recorder (RASP) setup.

The marine soundscape and fish biophony of the CCIP MPA were investigated between the 21^st^ and the 24^th^ of July 2020, under the permission of the MPA management body (N°103/2020). The CCIP MPA was established in 2002 with three zones of different protection levels including: Zone A-integral protection (only authorised scientific research is permitted), Zone B-partial protection, and Zone C-general protection (anchoring, mooring, and fishing activity are permitted but regulated). Within the whole CCIP MPA, three sampling sites were selected: Sant’Antonio (SA) inside the Zone A, Punta Giglio (PG) inside the Zone B, and Bramassa (BR) inside the Zone C (Fig. 2A). In each site, two underwater autonomous acoustic recorders (RASP-URec384k-Nauta RCS, here after RASP) were deployed at a distance of 200–600 m (area 1 and 2) and at a depth of about 10 m on a rocky bottom within small patches of the seagrass *Posidonia oceanica* (Table 1). RASP canisters were connected to ballast weights (10 kg) and a buoy (Fig. 2B) that were placed on the bottom *via* SCUBA. The days of deployment were characterized by good weather conditions, namely sea state < 2 (Douglas scale) and wind force < 2 (Beaufort scale), to reduce the influence of wind and waves on the marine soundscape.

**Table 1:** Sampling scheme.

Site	Area	MPA Zone	Coordinates (Lat-Long)	Depth (m)	Habitat	Date	Duty cycle
SA	1	A	40°34′09″N 8°13′45″E	10–15	R/P	July 22^-^23	24 min of recordings (2 min every 5 min) *per* hour, for 24 h
SA	2	A	40°34′09″N 8°14′00″E	10–14	R/P	July 23^-^24	
PG	1	B	40°34′13″N 8°12′28″E	12–15	R/P	July 23^-^24	
PG	2	B	40°34′16″N 8°12′35″E	12–15	R/P	July 22^-^23	
BR	1	C	40°35′02″N 8°12′13″E	7–8	R/P	July 21^-^22	
BR	2	C	40°35′54″N 8°12′07″E	7–8	R/P	July 22^-^23	

**Note:**

R, rocky reef; P, *Posidonia oceanica*; SA, Sant’Antonio; PG, Punta Giglio; BR, Bramassa.

Each RASP was featured with a Sensor Technology SQ26 pre-amplified hydrophone (Sensitivity-164 dB re: V/μ Pa, flat frequency response), a DODOTRONIC programmable recorder (gain 0 dB), battery set, and 400 Gb-SD memory card. The recorders were set to record a total of 24 h per deployment (Table 1) with a duty cycle of 2 min every 5 min, obtaining 24 min of recordings per hour at a sampling rate of 384 kHz (16 bit resolution) for a total of 1,152 min per site. Each collected file was further assigned to either day (from 7 am to 8 pm) or night (from 9 pm to 6 am) depending on the time of the recording. Dawn and dusk were attributed to the night-time.

A simultaneous study was conducted at the same sites by means of BRUV (Baited Remote Underwater Video) usage to determine the diversity of species and the abundance of fish (Manghi et al., 2020; La Manna et al., 2021). For each site, six BRUV units were deployed on the bottom, at a depth range of 7–15 m, between 9 am and 3 pm. A total of 300 min of video recordings per site were analyzed *via* the SeaGIS Event Measure software (www.seagis.com.au). Footage was analysed for the 50 min soak time, recording any identifiable species and MaxN (the maximum number of individuals observed in one single frame) every 30 s (Willis & Babcock, 2000).

### Acoustic analysis

Each 2 min recording was analyzed by means of the PAMGuide, a template code provided in R (Merchant et al., 2015) able to perform all the signal processing steps required for the calibration procedure to obtain absolute sea ambient noise (SAN) levels. For the purposes of this study, SAN was defined as “all sounds (both natural and anthropogenic) except for those resulting from the deployment or recovery of the recording equipment” (Robinson, Lepper & Hazelwood, 2014).

To characterize the contribution of both biophony and anthrophony, SAN levels were measured as sound pressure level (SPL dB re: 1 μ Pa-rms, hereafter “SPL”) at eight 1/3 octave bands, centred from 125 to 16 kHz (125, 250, 500, 1,000, 2,000, 4,000, 8,000, and 16,000 Hz).

Each recording was visually and aurally inspected by Raven Pro 1.5 (Cornell University), displaying the spectrogram 10 s at a time with frequencies between 0 and 8.5 kHz (FFT length = 8,192; Hamming window, 50% overlap). The presence of boat passages (counted as number of boats/minute), dolphins, and other biological signals (*e.g.*, snapping shrimps) were noted from the spectrogram in Raven.

Fish sounds were classified and counted following a catalogue of known sounds from the Mediterranean Sea (Picciulin et al., 2012; Bertucci et al., 2015; Desiderà et al., 2019) (Table 2; Material S1) based on the acoustic characteristics of the sound type. Due to the high presence of invertebrate acoustic signals, the loud boat noises and the poor signal to noise ratio of the majority of fish sounds, a more detailed characterization of the sound categories based on the measurements of sound duration, number of pulses, inter-pulses interval duration, and dominant frequencies was not possible. Thus, we distinguished between frequency modulated sound and series of pulses. In the first category, we considered a single sound or series of down-sweeping sounds (sounds with decreasing frequency) with a peak frequency ≤ 200 Hz (low frequency down-sweep, LDS, associated with *Epinephelus marginatus*, Bertucci et al., 2015) or ≥ 200 Hz (down-sweep, DS, and down-sweep series, DSS). In the second category, we considered (i) irregular series of at least three impulsive sounds with peak frequency ≤ 200 Hz (low-frequency pulse sequence, LPS, likely emitted by *Epinephelus marginatus*, Bertucci et al., 2015; low-frequency fast pulse train, LFPT; low-frequency down-sweep pulse series, LDSPS), (ii) irregular series of at least three impulsive sounds with peak frequency ≥ 200 Hz (pulse series, PS; fast pulse train, FPT), and (iii) regular series of at least three pulses (down-sweep series, DSS1; regular pulse series, RPS, associated with *Sciaena umbra*; alternating pulse period pulse series, APPPS, associated with *Ophidion rochei*; ultra-fast pulse series, UFPS/*kwa*, associated with *Scorpaena* spp.) (Table 2; Material S1). In detail, we classified (i) RPS, the sounds made up of four to seven low-frequency pulses with a pulse period of ca. 70–140 ms and a pulse duration of ca. 16–27 ms (Picciulin et al., 2012; Parmentier et al., 2018), (ii) APPPS, the sounds made up of long trains of about 40 low frequency pulses showing the typical and unique pulse period alternation pattern characterising the *O. rochei* calls (Kéver et al., 2012; Picciulin et al., 2018a), (iii) UFPS “kwa”, the sound composed of about 13 pulses with 13 ms intervals and average dominant frequency at about 750 Hz (Di Iorio et al., 2018; Bolgan et al., 2019) (Material S1).

**Table 2:** Sound categories considered in the analysis, modified by Desiderà et al. (2019).

Sound category	Name	Definition (references)	Associated species
**Frequency modulated**			
Low frequency down-sweep	LDS	Down-sweep frequency modulated sound with peak frequency <200 Hz (Bertucci et al., 2015)	*Epinephelus marginatus*
Down-sweep	DS	Down-sweep frequency modulated sound (Desiderà et al., 2019)	Unknown
Down-sweep series	DSS	Consecutive down-sweeps modulated sound (Desiderà et al., 2019)	Unknown
Long tonal call	LT	Long tonal call (about or more than 1 s) with no frequency modulation (Desiderà et al., 2019)	Unknown
**Irregular pulse series**			
Low frequency pulse series	LPS	Sequence of pulses (at least three). Irregular pulse period. Bandwidth between 20 and 200 Hz. Peak frequency < 200 Hz (Desiderà et al., 2019; Bertucci et al., 2015)	*Epinephelus marginatus*
Low frequency fast pulse train	LFPT	Pulse train with short and almost undetectable pulse train. Peak frequency < 200 Hz (Desiderà et al., 2019)	Unknown
Low frequency down-sweep pulse series	LDSPS	Consecutive down-sweeps and pulses. Peak frequency < 200 Hz (Desiderà et al., 2019)	Unknown
Pulse series	PS	Sequence of pulse (at least three). Irregular pulse period. Peak frequency > 200 Hz (Desiderà et al., 2019)	Unknown
Fast pulse train	FPT	Pulse train with short and almost undetectable pulse train. Peak frequency > 200 Hz (Desiderà et al., 2019)	Unknown
**Regular/stereotyped pulse series**			
Down-sweep series	DSS	Series of consecutive down-sweeps	Unknown
Regular pulse series	RPS	Stereotyped pulse series (Picciulin et al., 2012)	*Sciaena umbra*
Pulse series with alternating pulse period	APPPS	Stereotyped accelerating pulse series with alternating pulse period (Kéver et al., 2012)	*Ophidion rochei*
Ultra-fast pulse series	UFPS (kwa)	Frequency around 800 Hz. Pseudo-harmonics appearance in the spectrogram (Di Iorio et al., 2018; Desiderà et al., 2019).	*Scorpaen*a spp.

### Statistical analysis

Temporal and spatial trends of SAN levels and boat passages were investigated, testing each octave band’s SPLs and the number of boats/minute as a function of the site (SA, PG, BR), time (day and night) and hour (24 h) with the Kruskal-Wallis test or the Mann Whitney U test, since the data distributions were not normal.

The frequency of occurrence of each identifiable biological sound, mainly fish sound type, was calculated as the percentage of any sound type over the total sound types counted hourly. From the number of fish sounds by type, the following variables were calculated (i) sound type richness, expressed as the number of different sound type categories found per hour of recording, (ii) abundance, expressed as the number of sounds associated to each of the above described sound type categories per hour of recording, (iii) Shannon-Wiener diversity index as described by Barnes et al. (1998), which combines sound type richness (the number of sound type per hour of recording) and their relative abundance.

The differences in the values of these three variables as a function of site (SA, PG, BR), time (day and night), and hour (24 h) were explored with the Kruskal-Wallis test or the Mann Whitney U test, after testing for normality of data distribution with the Shapiro-Wilk test and heterogeneity with the Bartlett test. A pairwise Wilcox test was run to compare group levels. A non-metric multidimensional ordination (nMDS) was produced from the sample similarity matrix to visually represent the similarity of fish biophony between site and time. Values were fourth-root transformed before calculating the Bray-Curtis similarity. A one-way non-parametric similarity analysis (ANOSIM) was applied on the same matrix to test the null hypothesis stating that there was no difference in fish biophony across site and time levels. ANOSIM statistic calculates the ratio (R) between the mean of ranked dissimilarities between groups and the mean of ranked dissimilarities within groups. The “kwa” sounds (Di Iorio et al., 2018; Bolgan et al., 2019) were removed from the latter analysis to reduce the influence of these largely dominant sounds (Guidetti & Sala, 2007). *p* values were considered significant at *p* < 0.05. All statistical analyses were carried out in R (R Core Team, 2015) using the packages ‘vegan’ (Oksanen, 2017) and ‘MASS’ (Venables & Ripley, 2002).

## Results

**Figure 3:**
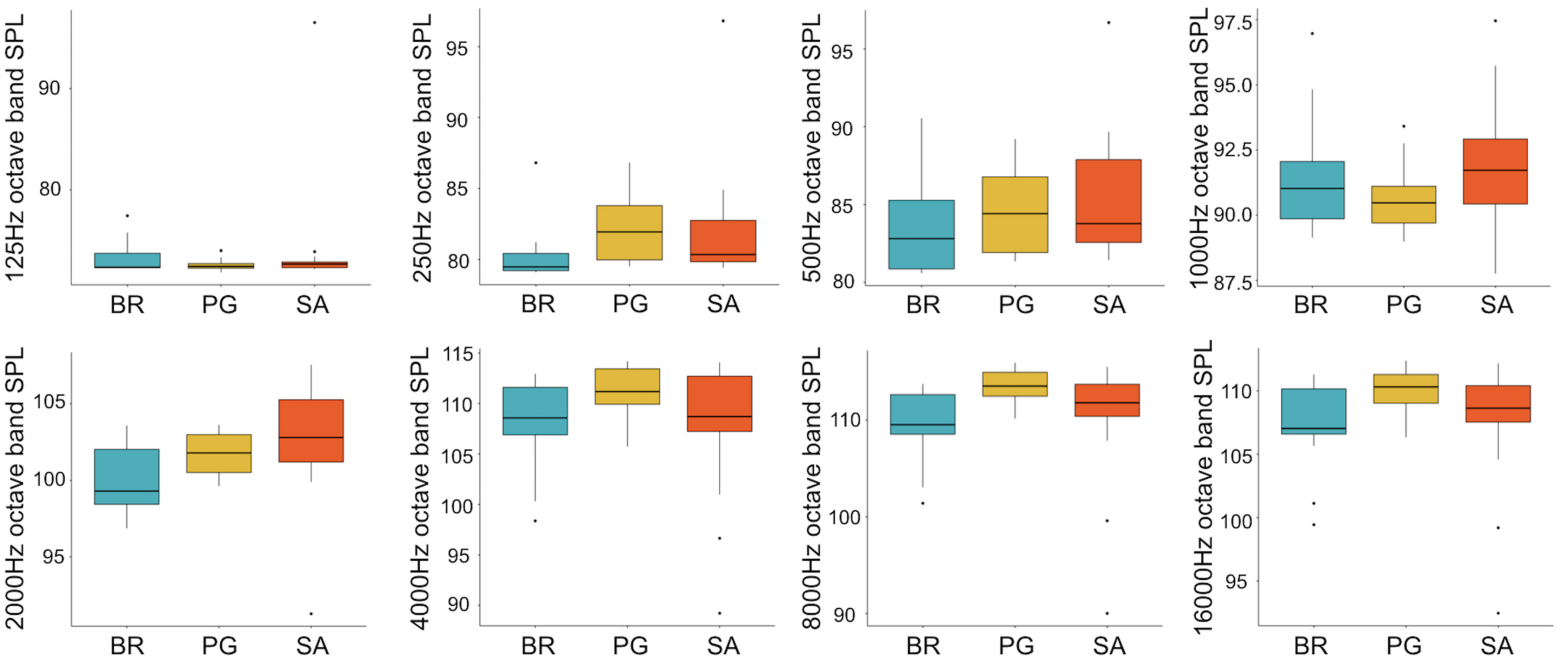
Boxplot of the octave band SPLs (dB re 1 µPa) as a function of site. BR, Bramassa; SA, Sant’Antonio; PG, Punta Giglio. The thick black lines represent the medians, the boxes encompass the 25% and 75% quartiles, the whiskers extend to the most extreme data points within 1.5× the interquartile range outside the box, and the circles show data points beyond the whiskers.

**Figure 4:**
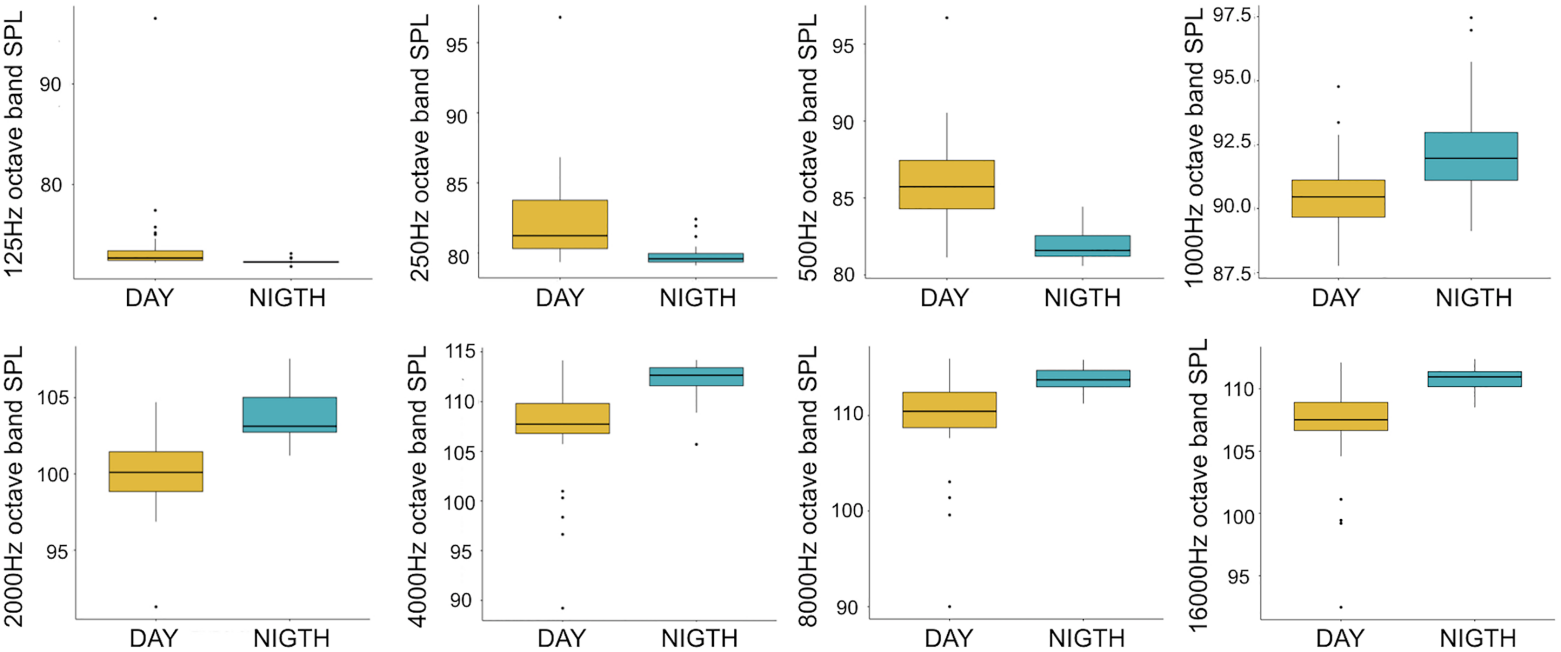
Boxplot of the octave band SPLs (dB re 1 µPa) as a function of time (day and night). The thick black lines represent the medians, the boxes encompass the 25% and 75% quartiles, the whiskers extend to the most extreme data points within 1.5× the interquartile range outside the box, and the circles show data points beyond the whiskers.

**Figure 5:**
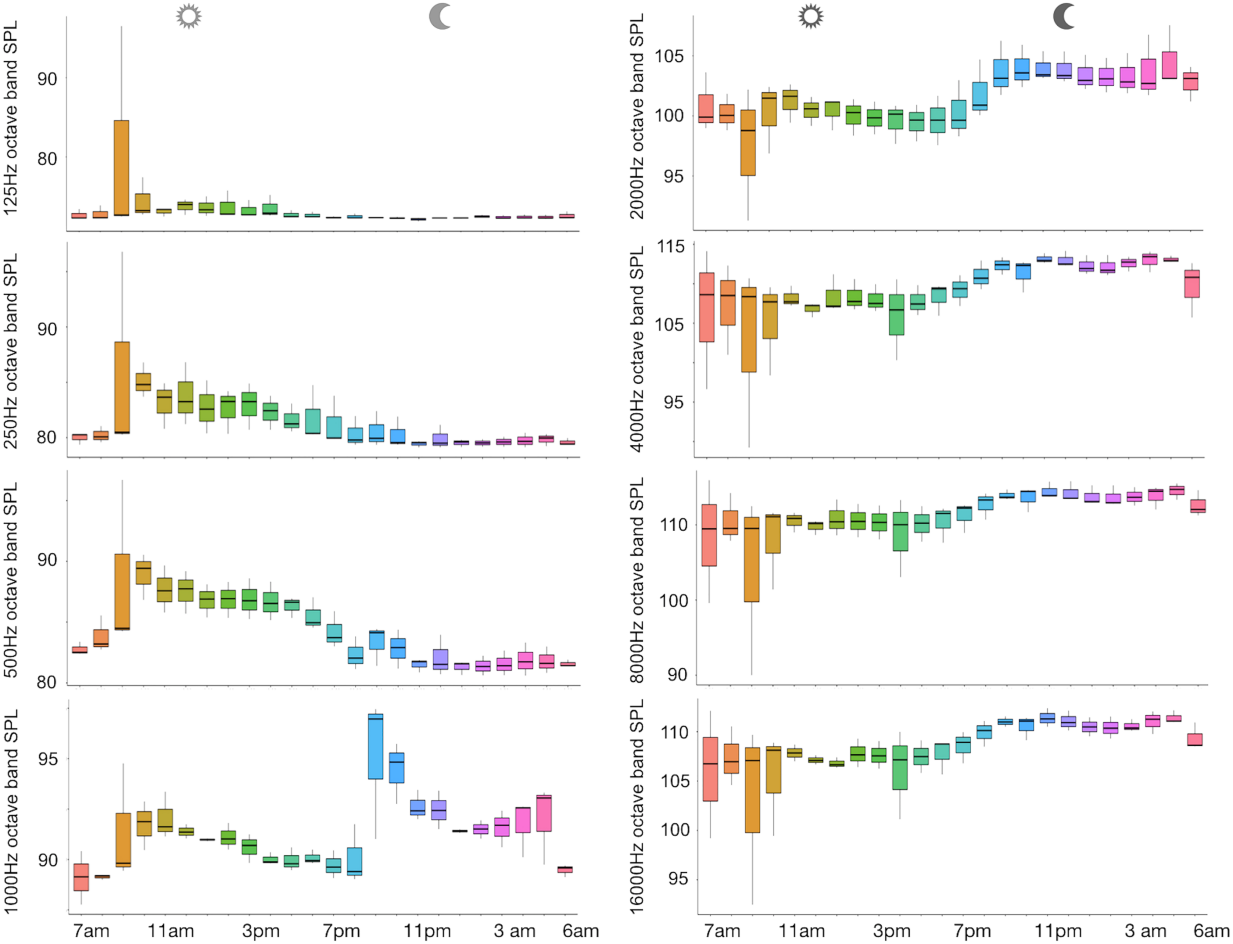
Boxplot of the octave band SPLs (dB re 1 µPa) as a function of hour. The thick black lines represent the medians, the boxes encompass the 25% and 75% quartiles, the whiskers extend to the most extreme data points within 1.5× the interquartile range outside the box, and the circles show data points beyond the whiskers.

**Figure 6:**
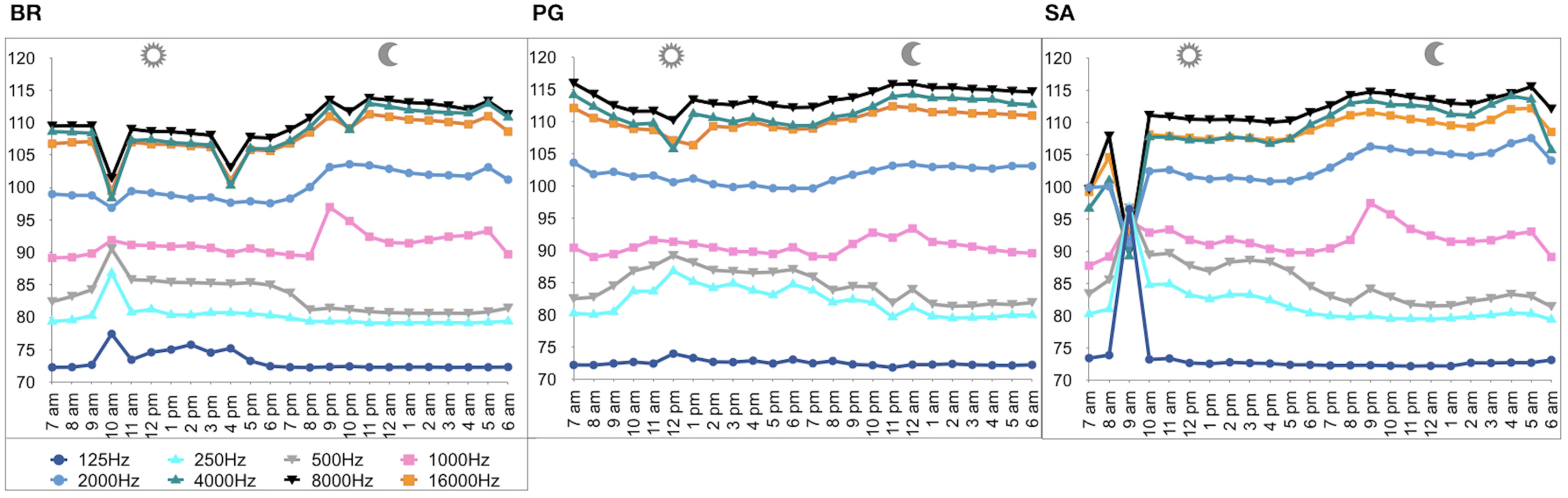
SPLs (dB re 1 µPa) hourly trend in the three sites. (BR, Bramassa; SA, Sant’Antonio; PG, Punta Giglio).

A total of 3,456 min of recordings were collected and SAN levels were measured as octave band SPLs (dB re: 1 μ Pa-rms). The band centred at 125 Hz ranged from 72 to 95 dB, with no difference between sites, while all the other octave band SPLs were site dependent (Table 3, Fig. 3). The octave band SPLs centred at 250, 500, and 1,000 Hz were between 79 and 97 dB, while the higher bands were between 90 and 116 dB (Fig. 3). The bands centred at 250, 500, 4,000, 8,000, and 16,000 Hz were higher at PG (Zone B) compared to SA (Zone A) and BR (Zone C), while octave band SPLs centred at 1,000 and 2,000 Hz were higher at SA compared to PG and BR (Table 3, Fig. 3). Octave band SPLs centred at 125, 250, and 500 Hz were on average between 1 dB and 4 dB louder during the day than at night. All the other octave band SPLs were on average between 1.5 dB and 4.5 dB louder at night than during the day (Table 3), particularly from 9 pm to 5 am (Table 3, Figs. 4–6).

**Table 3:** Results of the Kruskal–Wallis and Mann Withney U tests on the octave band SPLs and the mean number of boat/min as function of site, time and hour.

	SITE			TIME		HOUR		
	KW	df	*p*-value	W	*p*-value	KW	df	*p*-value
125 Hz SPL	2.2468	2	0.3252	1,036	<0.0001	39.731	23	0.0165
250 Hz SPL	14.841	2	0.0006	1,112	<0.0001	45.196	23	0.0038
500 Hz SPL	7.3198	2	0.02573	1,181	<0.0001	56.632	23	0.0001
1,000 Hz SPL	6.528	2	0.03824	279	<0.0001	47.962	23	0.0017
2,000 Hz SPL	13.747	2	0.0010	79	<0.0001	44.091	23	0.0051
4,000 Hz SPL	9.555	2	0.0084	112	<0.0001	44.403	23	0.0047
8,000 Hz SPL	20.23	2	<0.0001	126	<0.0001	38.865	23	0.02053
16,000 Hz SPL	12.172	2	0.0022	102	<0.0001	44.253	23	0.004
Boat/min	10.343	2	0.0056	4,529	0.001	95.604	23	<0.0001

**Note:**

BR, Bramassa; PG, Punta Giglio; SA, Sant’Antonio.

### Biophony and anthrophony

The soundscape was dominated by the impulsive sounds related to snapping shrimps and other invertebrates (present in 98% of the recordings), fish sounds, and boat passages. Dolphins were only present in five recordings (at BR and SA).

**Figure 7:**
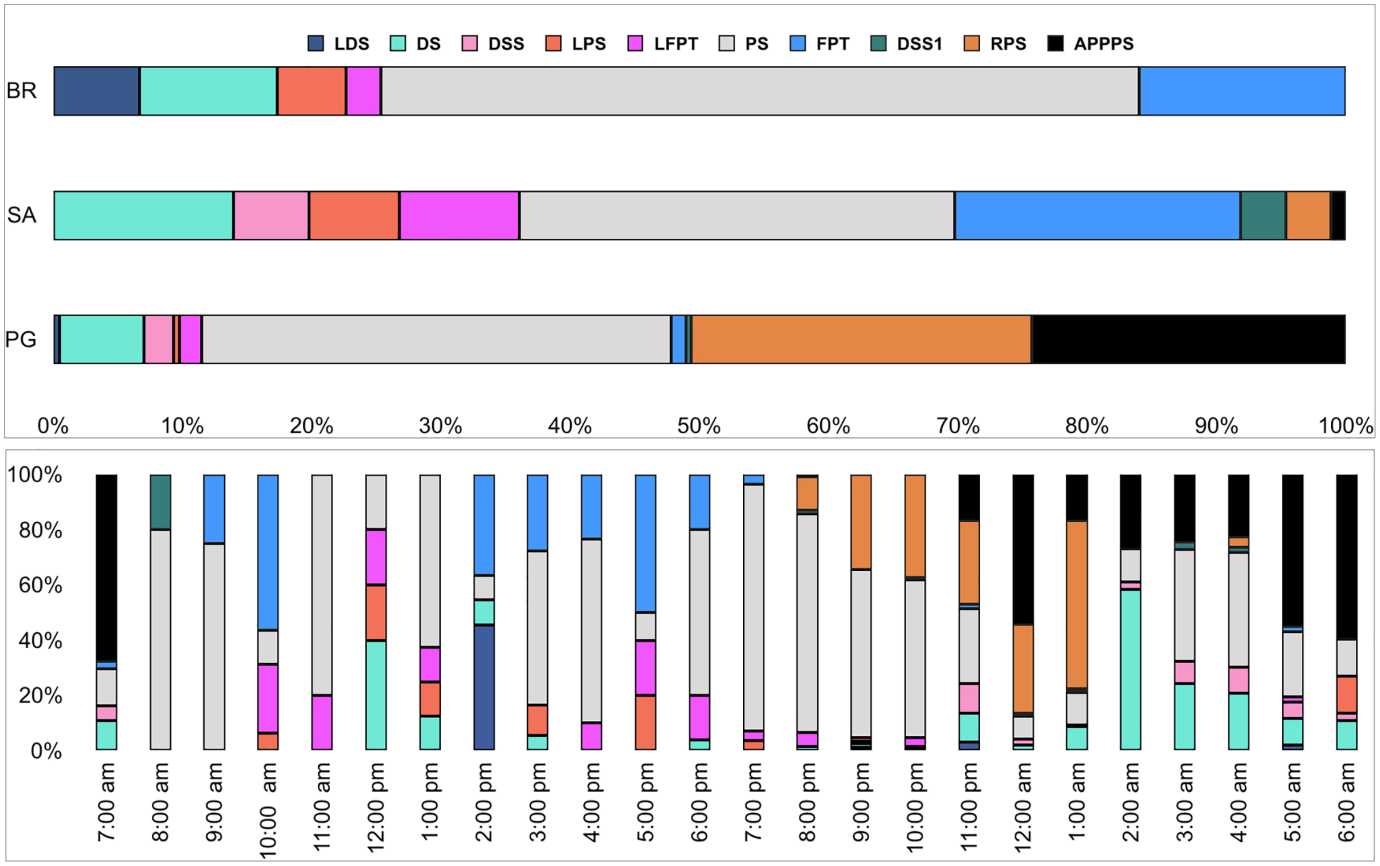
Fish sound occurrence (%) in the three sites and as a function of hour. BR, Bramassa; SA, Sant’Antonio; PG, Punta Giglio.

**Figure 8:**
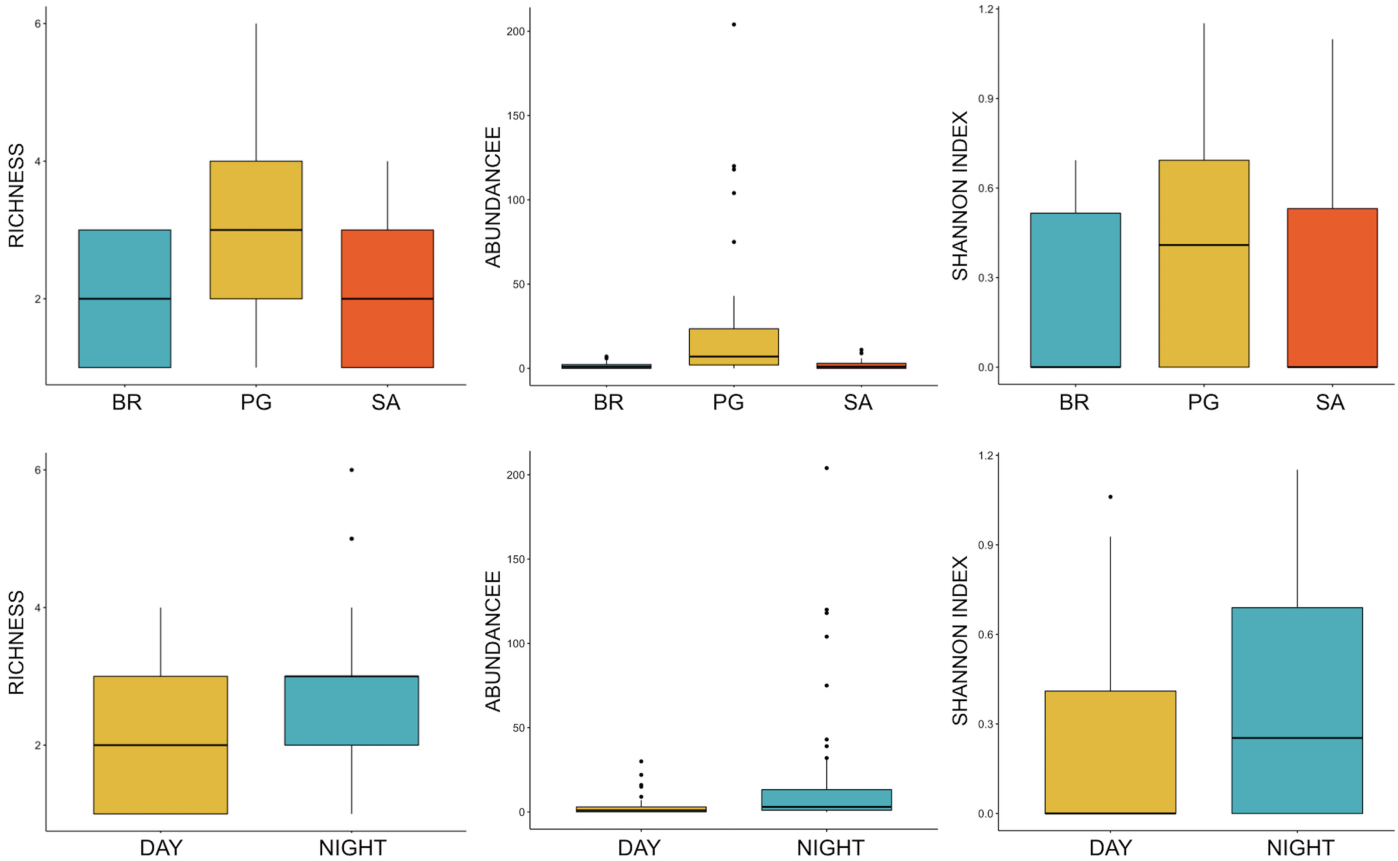
Boxplot of richness, abundance and Shannon Index as a function of site and time. BR, Bramassa; SA, Sant’Antonio; PG, Punta Giglio. The thick black lines represent the medians, the boxes encompass the 25% and 75% quartiles, the whiskers extend to the most extreme data points within 1.5× the interquartile range outside the box, and the circles show data points beyond the whiskers.

**Figure 9:**
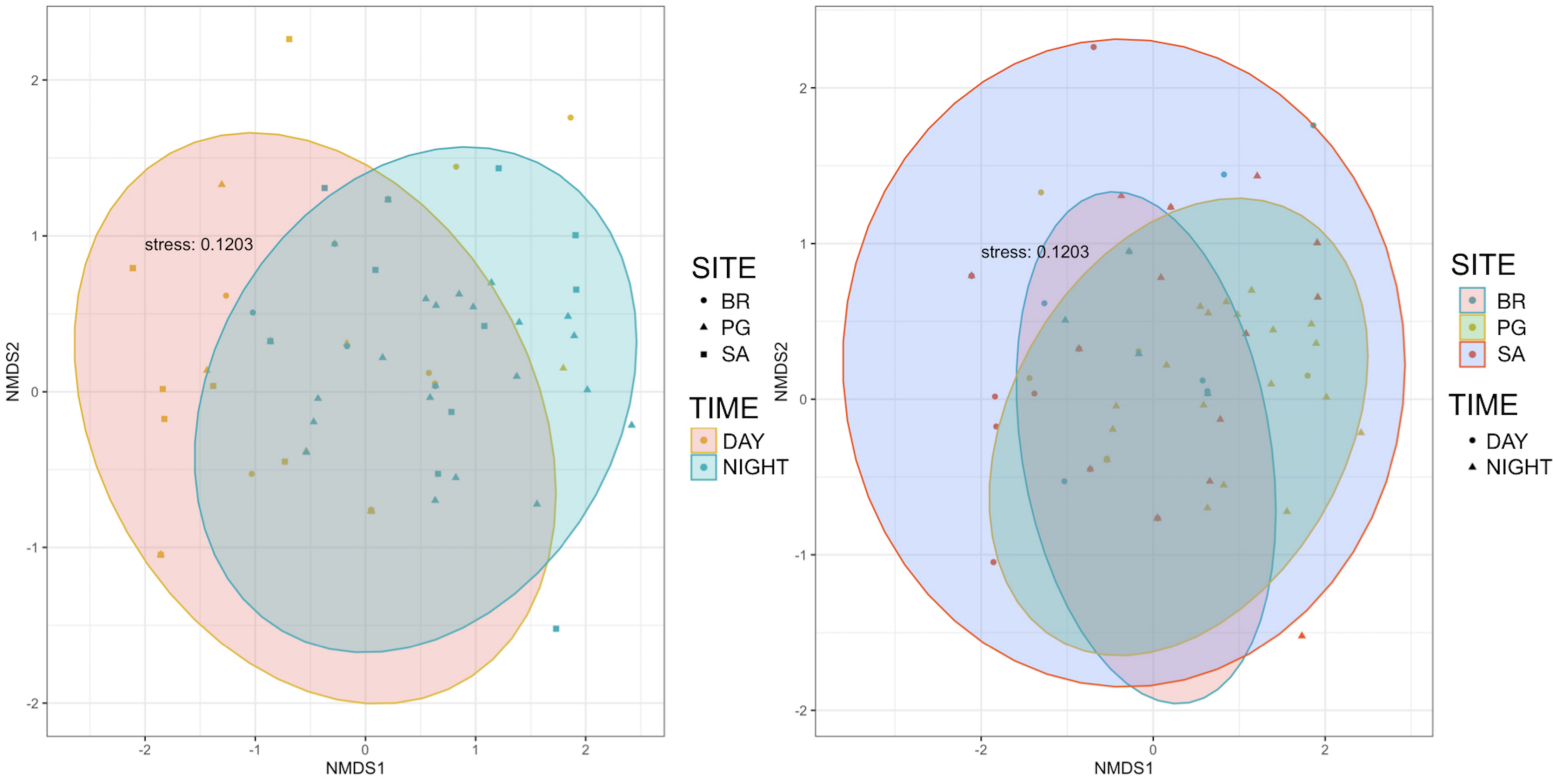
Multidimensional scaling plots showing the similarity of species composition grouped by site and time. BR, Bramassa; SA, Sant’Antonio; PG, Punta Giglio.

Ten of the 12 sounds from the Mediterranean fish sound catalogue were recorded. The “kwa” was the most abundant sound at any site (over 47,596 sounds) and mainly at night (85%) rather than during the day. After the “kwa” sound, PS, RPS, APPPS, and DS had the highest occurrence, while all the other sounds were recorded in a percentage lower than 3% (Table 4, Fig. 7). The percentage of fish sound occurrence was site dependent, and interestingly, the sounds recorded at PG accounted for 87% of the total sounds (Table 4, Fig. 7). Sound type diversity was strongly time dependent as some were recorded exclusively during nocturnal (*e.g*., RPS, APPPS, DSS) or diurnal hours (*e.g*., LPS, LFPT, FPT), while only two sound types were recorded during both night and day (*e.g*., PS, DS) (Fig. 7). Sound type richness was different as a function of site and time while no differences were found as a function of hours (Table 5, Fig. 8). Particularly, richness was higher at night than during the day and in PG compared to SA and BR. The same trend was also true for the abundance and the Shannon-Wiener index, which were both higher in PG and at night (Fig. 8), while no differences were found across hours. The differences found were also evidenced by the nMDSs and the ANOSIMs which highlighted different fish biophony depending on the site (R = 0.1205, *p*-value = 0.001) and time (R = 0.06615, *p*-value = 0.001) (Fig. 9).

**Table 4:** Number and percentage of the sound types recorded in each site and all sites combined.

Sound type	All SITES	SA (A ZONE)	PG (B ZONE)	BR (C ZONE)
	*N* (%)	*N* (%)	*N* (%)	*N* (%)
LDS	10 (1%)	0 (0%)	5 (0.5%)	5 (7%)
DS	92 (7%)	12 (14%)	72 (7%)	8 (11%)
DSS	30 (2%)	5 (6%)	25 (2%)	0 (0%)
LPS	15 (1%)	6 (7%)	5 (0.5%)	4 (5%)
LFPT	29 (2%)	8 (9%)	19 (2%)	2 (3%)
PS	472 (37%)	29 (34%)	399 (36%)	44 (59%)
FPT	44 (3%)	19 (22%)	13 (1%)	12 (16%)
DSS1	7 (1%)	3 (3%)	4 (0.4%)	0 (0%)
RPS	293 (23%)	3 (3%)	290 (26%)	0 (0%)
APPPS	268 (21%)	1 (1%)	267 (24%)	0 (0%)
**TOTAL**	1,260 (100%)	86 (7%)	1,099 (87%)	75 (6%)
UFPS (kwa)	47,596 (100%)	11,656 (24%)	16,341 (34%)	19,600 (41%)

**Note:**

SA, Sant’Antonio; PG, Punta Giglio; BR, Bramassa.

**Table 5:** Results of the Kruskal-Wallis and Mann Withney U tests on the sound richness, abundance and Shannon Weiner Index as function of site, time and hour.

	SITE			TIME		HOUR		
	KW	df	*p*-value	W	*p*-value	KW	df	*p*-value
**Sound Richness**	17.645	2	0.001	1,738	0.001	17.28	23	0.7953
**Abundance**	32.423	2	<0.0001	1,715	<0.001	27.441	23	0.2377
**Shannon Wiener Index**	12.402	2	0.002	1,892	0.002	25.517	23	0.3242

**Note:**

BR, Bramassa; PG, Punta Giglio; SA, Sant’Antonio.

A visual fish census was conducted simultaneously using a BRUV and recorded 1,877 individuals (abundance expressed as MaxN) belonging to 38 taxa and 13 families. In particular, 28 taxa and 657 individuals were observed at SA (Zone A), 30 taxa and 888 individuals at PG (Zone B) and 26 taxa and 332 individuals at BR (Zone C) (Table 6). The obtained results confirmed the outputs from the acoustic monitoring. PG was the site with the highest number of species and abundance of individuals compared to BR and SA.

**Table 6:** MaxN of each species observed by BRUV.

FAMILY	SPECIES	BR	PG	SA
Apogonidae	*Apogon imberbis*	3	1	1
Blennidae	*Parablennius rouxi*	1	1	1
Gobiidae	*Gobius cruentatus*	1	0	0
Gobiidae	*Gobius marmoratus*	0	0	0
Labridae	*Coris julis*	26	18	18
Labridae	*Crenilabrus melanocerus*	7	2	3
Labridae	*Labrus merula*	0	0	2
Labridae	*Labrus viridis*	1	1	1
Labridae	*Symphodus cinereus*	1	1	0
Labridae	*Symphodus doderleini*	8	5	1
Labridae	*Symphodus ocellatus*	5	1	0
Labridae	*Symphodus roissali*	0	0	9
Labridae	*Symphodus rostratus*	2	0	1
Labridae	*Symphodus tinca*	9	10	12
Labridae	*Thalassoma pavo*	3	7	9
Mugilidae	Mugilidea spp	1	0	11
Mullidae	*Mullus surmuletus*	12	15	0
Pomacentridae	*Chromis chromis*	115	517	412
**Sciaenidae**	***Sciaena umbra***	**0**	**6**	**0**
**Scorpaenidae**	***Scorpaena maderensis***	**0**	**1**	**0**
**Scorpaenidae**	***Scorpaena notata***	**0**	**0**	**1**
Serranidae	*Dicentrarchus labrax*	0	3	0
**Serranidae**	***Ephinephelus marginatus***	**1**	**1**	**0**
Serranidae	*Serranus cabrilla*	5	2	6
Serranidae	*Serranus scriba*	6	4	2
Sparidae	*Diplodus annularis*	30	13	2
Sparidae	*Diplodus vulgaris*	13	97	103
Sparidae	*Diplodus sargus*	29	35	18
Sparidae	*Diplodus puntazzo*	0	1	5
Sparidae	*Diplodus cervinus*	0	1	0
Sparidae	*Sparus aurata*	1	6	2
Sparidae	*Dentex dentex*	0	1	1
Sparidae	*Oblada melanura*	25	46	17
Sparidae	*Salpa salpa*	22	85	29
Sparidae	*Seriola dumerili*	0	3	3
Sparidae	*Spondyliosoma cantharus*	2	1	1
Sphyraenidae	*Sphyraena viridensis*	1	3	3
Trypterygiidae	*Trypterygion spp*	0	0	1
**TOTAL FISH COUNT**		**332**	**888**	**675**

**Note:**

BR, Bramassa (C zone); PG, Punta Giglio (B zone); SA, Sant’Antonio (A zone). In bold the vocal species.

**Figure 10:**
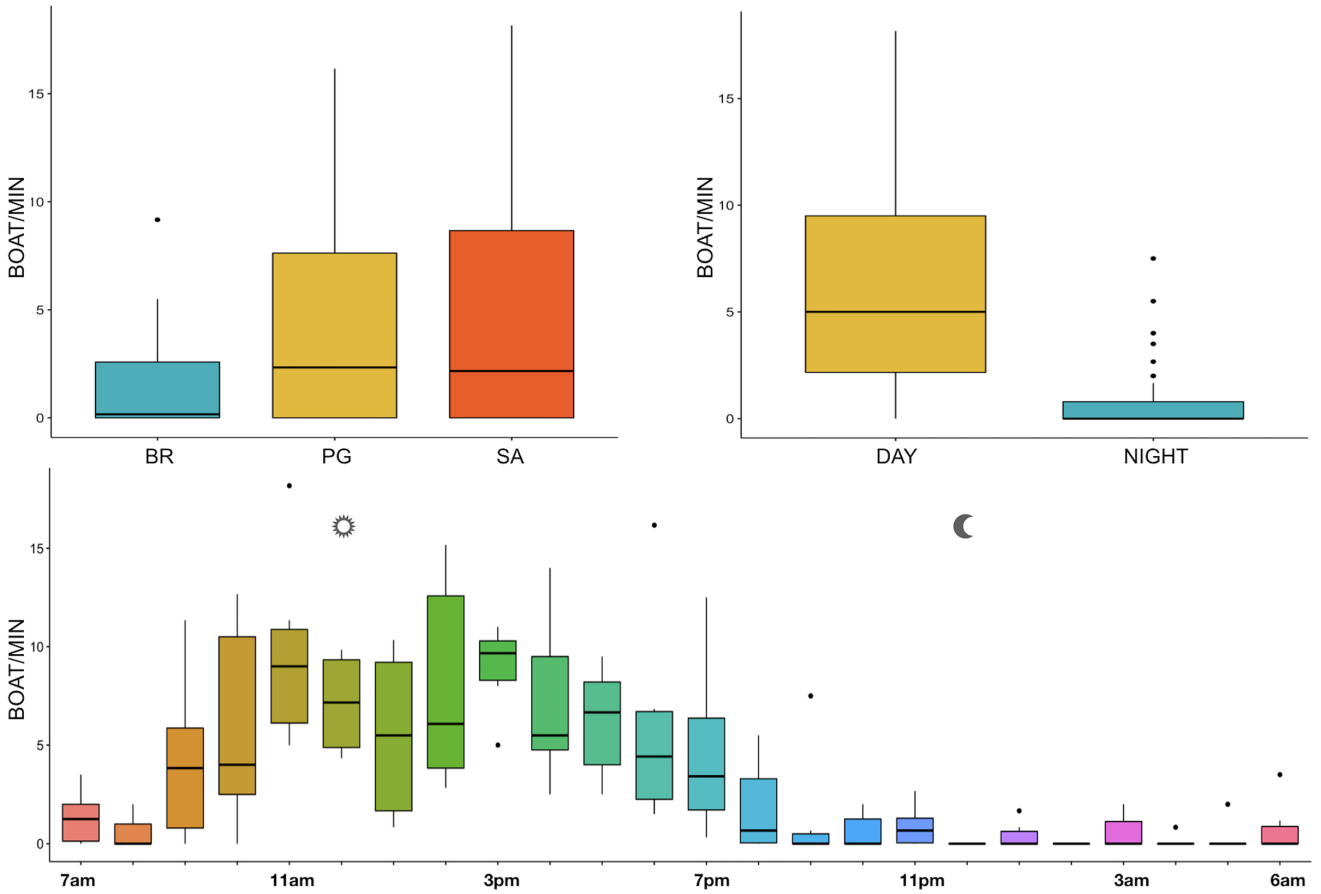
Boxplot of number of boat/min as a function of site, time and hour. BR, Bramassa; SA, Sant’Antonio; PG, Punta Giglio. The thick black lines represent the medians, the boxes encompass the 25% and 75% quartiles, the whiskers extend to the most extreme data points within 1.5× the interquartile range outside the box, and the circles show data points beyond the whiskers.

The noise from boat passages was the only component of anthrophony detected. The mean number of boats/minute was higher at PG (Zone B) and SA (Zone A) than at BR (Zone C) and during the day than at night, mainly from 9 am to 7 pm (Table 3, Fig. 10).

## Discussion

This study has characterised the CCIP MPA soundscape and disentangled some biological and anthropogenic components providing evidence that patterns of variability are strongly related to the time of day and the sites within the MPA, despite the narrow time interval (1 day) and short distances (few kms) investigated.

Since RASP deployments occurred only in calm sea and low wind conditions, no significant inputs from geophonies can be responsible for the SPLs at low frequencies (Haxel, Dziak & Matsumoto, 2013), with the only exception of the waves generated by boats. SPLs centred at 125, 250, and 500 Hz were significantly higher in the daytime, due to the high number of boats per minute whose noise dominated the soundscapes (refer to Fig. 4). Interestingly, between 9 am and 10 am at SA, the site with the highest number of boat passages, SPLs at low frequencies (between 125 and 500 Hz) increased from 10 dB to 20 dB above the sea ambient noise levels of the preceding or following hours (refer to Fig. 6). This trend was also found, but less prominent, at the other two sites and in correspondence with the day-time when ferries and excursion boats depart from the port of Alghero to reach the tourist destinations within the CCIP MPA (La Manna *pers. obs*.). The loudest man-made noise was found in the Zone A, followed by B, and then the Zone C, showing that CCIP MPA restrictions do not provide sufficient protection from acoustic pollution (La Manna et al., 2016). This is not surprising since the extension of the fully protected zone is extremely limited and cannot provide any barrier to the propagation of vessels’ sounds that can navigate on the edge of its boundaries (Buscaino et al., 2016), while in the other zones, navigation is not restricted at all. Together with the maximum increase in the low frequency noise (from 9 am to 10 am), a reduction of the SPLs between 2 and 16 kHz was also observed. Further studies are needed to verify if this reduction may be related to the interruption or decrease in the production of biological sounds as a consequence of the masking noise produced by boats.

The dominant biological components of the CCIP MPA soundscape identified by the spectrogram analysis were the impulsive sounds likely generated by some invertebrates and the snapping shrimps of the family Alpheidae. *Alpheus* spp. usually produces broadband pulses with most energy peaks between 2 and 4 kHz (McWilliam & Hawkins, 2013), while *Athanas nitescens*’ signals have a peak frequency in the range between 5 and 11 kHz (Coquereau et al., 2016). Even if no data are available about the presence of these invertebrates in the CCIP MPA, the increase of SPL levels at the octave bands above 2 kHz (see also Materials S2), mainly during the nocturnal hours, is consistent with the reported acoustic properties and daily rhythms of snapping shrimp sound in the Mediterranean (Buscaino et al., 2016; Pieretti et al., 2017) and other regions (Radford et al., 2008b; Radford et al., 2010; Lillis & Mooney, 2018). Another potential source of biological sounds were the grazing activities of sea urchins (*Paracentrotus lividus* and *Arbacia lixula*). The spectrogram analysis was unable to distinguish the sounds related to sea urchins from the impulsive sounds of snapping shrimps and other invertebrates, however, the relative frequencies partially overlapped (Au & Banks, 1997; Radford et al., 2008a). The importance of sea urchin chorus in the characteristics of rocky reef soundscapes needs to be deeper investigated. The dramatic reduction of the sea urchin stock in Alghero, and more generally throughout Sardinian and other Mediterranean regions (Coppa et al., 2021; Ceccherelli, Pinna & Pansini, 2019), has brought about the question of the effect that the low abundance of adult sea urchins can have on the marine soundscape, as well as on the recruitment for the known ability to acoustically direct larvae movement and provide metamorphosis signals, similarly to fish and other invertebrate species larvae (Tolimieri et al., 2004; Jeffs, Montgomery & Tindle, 2005; Radford, Jeffs & Montgomery, 2007; Simpson et al., 2008).

The bands centred at 1,000 Hz, and marginally at 2,000 Hz, are those particularly affected by the presence of fish sounds (refer to Figs. 3–5). Acoustic analysis detected 10 of the 12 sounds classified for the Mediterranean Sea by Desiderà et al. (2019). Excluding the ubiquitous “kwa”, the most frequent sound type was PS (recorded in all sites with percentages of occurrence between 34% and 59%) (refer to Fig. 7). This category of sound, whose species emitters are still unknown, deserves further investigation given the high intra-class variability of the acoustic features observed.

The vast majority of fish sounds were recorded at PG, which was the site with the highest sound richness, abundance, and Shannon-Wiener index (refer to Fig. 8). Interestingly, visual census data collected simultaneously using BRUV, supported the idea that the acoustic diversity reflects the local fish biodiversity. More in detail, the visual census based on BRUV (see also Manghi et al., 2020; La Manna et al., 2021) and Underwater Visual Census (UVC) (Grech et al., 2020) techniques detected *Sciaena umbra* at PG only once for a maximum number of 14 individuals, thus indicating a local low density of this species. The acoustic monitoring confirms that the species’ distribution was almost exclusively confined at PG (only few *S. umbra* sounds have been recorded at SA). Although *S. umbra* sounds are produced by fish engaged in reproductive behaviours, the local spawning activity can be only hypothesised, lacking its typical acoustic proxy (*i.e.*, the chorus pattern) (Picciulin et al., 2018b). Acoustic monitoring also detected the sounds associated with species such as *Scorpaena* spp. and *Epinephelus marginatus* in sites (SA and BR) where they have not been detected by UVC (Grech et al., 2020), providing evidence of the usefulness of passive acoustic monitoring as a complementary technique of species census (Desiderà et al., 2019). Furthermore, at PG, the acoustic monitoring detected the sound associated with a cryptic species (APPPS), *Ophidion* spp., a fish typical of sandy bottom environments which move to higher sandy and rocky seafloor or *Posidonia oceanica* habitat (Keskin, 2007) during the night. Due to the cryptic behavior of this species, the traditional fish visual census can likely underestimate its occurrence and, in fact, the CCIP MPA species lists have never included it, similarly to other MPAs (*e.g.*, Miramare MPA at Trieste, Northern Adriatic Sea; Picciulin et al., 2018a). Therefore, our findings represent an important contribution to the monitoring of cryptic and vulnerable species of which little data are available.

With regard to the circadian rhythm of fish sound emissions, the present study confirmed the known, predominantly nocturnal, acoustic pattern of some species such as *Sciaena umbra* (Picciulin et al., 2012), *Ophidion* spp. (Kéver et al., 2016; Parmentier et al., 2010), *Scorpaena* spp. (Di Iorio et al., 2018; Bolgan et al., 2019; Desiderà et al., 2019), and the majority of the other acoustic signals whose emitters are unknown (Buscaino et al., 2016). In addition, some differences were found between daytime and nightime fish biophony (refer to Fig. 10), likely indicating a different acoustic behaviour of the emitting species, which would also need further investigations.

Acoustic cues have fundamental importance in fish and marine mammal behaviour, reproduction, and social relationship (Erbe, 2012; Popper & Hawkins, 2011), in fish and invertebrate larval settlement (Slabbekoorn & Bouton, 2008) and in the mediation of predator-prey interactions (Wale, Simpson & Radford, 2013; Sarà et al., 2007; La Manna et al., 2016). A large amount of evidence has already suggested that the continuous noise from ships and boats can reduce the fitness at individual and population level (Rolland et al., 2012; Williams et al., 2014), but also may impede trophic interactions, with fatal consequence for marine ecosystems (Popper & Hawkins, 2011). Waiting for technologies that can reduce noise emissions from ships and boats, despite the limitations due to the small size, MPAs should provide protection from ocean noise (Dolman, 2007) through the adoption of appropriate management actions, such as imposing a limited number of boats allowed to enter into the MPA, reducing the speed of boat navigation, modulating the access to important areas because of the occurrence of feeding or breeding species, and interdicting the navigation during periods of species vulnerability.

## Conclusions

In the present study, the spatial and temporal variation of the CCIP MPA soundscape was investigated with the aim of satisfying the requirements of the SCI “IT 01B010042” Management Plan to monitor underwater noise and boat traffic. The study was conducted in the light of some scientific evidence that had already demonstrated the negative impact of noise on some protected species (La Manna et al., 2016, 2019). Moreover, the study provided the first baseline data on SAN levels, their daily trend, and main anthropogenic and biological sources that form the local soundscape, highlighting significant differences between hour of day and site, despite the recordings having been conducted on a reduced spatial (few kms) and temporal scale (1 day). The soundscape in the daytime was dominated by boat noise as showed by the significant increase of SPLs at the bands centred at 125, 250, and 500 Hz. Even if the actual MPA regulation already provides boat speed limits and some access limitation, these are not adequate to protect marine species from acoustic pollution. In fact, the loudest man-made noise was found in the Zone A, the integral protection zone. These results are therefore fundamental for the MPA management and can lead to more restrictive regulations regarding boat traffic and the related noise.

Furthermore, this study demonstrated the presence of a species never before recorded in the MPA (*Ophidion* sp.) and clarified presence and distribution of some protected species, such as *Sciaena umbra* and *Epinephelus marginatus*, highlighting the effectiveness of PAM as a complementary technique to fish visual census. Data obtained through the coupling of different techniques (visual and acoustic sampling) can provide more exhaustive information on the structure and distribution of fish assemblages and their reproductive sites, satisfying some fundamental requirements of MPAs including detailed inventory of the fish biodiversity and the evaluation of the response to the protection of different groups/species (La Manna et al., 2021). In spite of that, these results should be considered preliminary. Monitoring should be continued annually and extended temporally and spatially with the aim to investigate the variability in the soundscape linked to both natural phenomena (*e.g.*, seasonal pattern, type of habitat) and fluctuations in anthropogenic activities, mainly tourism trends.

## Supplemental Information

10.7717/peerj.12551/supp-1 Supplemental Information 1 Spectrograms of the 10 occurring sounds identified as fish calls. LDS: Low frequency down-sweep; DS: Down-sweep; DSS: Down-sweep series; LPS: Low frequency pulse series; LFPT: Low fre-quency fast pulse train; PS: Pulse series; FPT: Fast pulse train; DSS: Down-sweep series; RPS: Regular pulse series; APPPS: Pulse series with alternating pulse period; UFPS (kwa): Ultra-fast pulse series. (FFT: 8192; Hamming window, 50% overlap)

10.7717/peerj.12551/supp-2 Supplemental Information 2 Spectral probability density. Example of spectral probability densities over 1 h, in the range 100 to 16,000 kHz, at 10 am and 12 am. BR: Bramassa; PG: Punta Giglio; SA: Sant’Antonio.

10.7717/peerj.12551/supp-3 Supplemental Information 3 Fish abundance and noise measurements. The number of fish sounds per hour, hourly abundance, richness and shannon index as a function of site, time and hour and Sound Pressure Levels in the 8 octave bands (from 125 Hz to 16 kHz) as a function of site, time and hour.

## Additional Information and Declarations

### Field Study Permissions

The following information was supplied relating to field study approvals (*i.e*., approving body and any reference numbers): Field experiments were approved by Capo Caccia Isola Piana Marine Protected Area (Determinazione n. 103 del 21/04/2020).

### Data Availability

The following information was supplied regarding data availability: The raw data are available in the Supplemental Files.
