# Supplementary material for: Marine soundscape and fish biophony of a Mediterranean marine protected area
Source: PeerJ. 2021 Dec 15;9:e12551. doi: 10.7717/peerj.12551 (PMC8684326; doi:10.7717/peerj.12551)
Supplement: Supplemental Information 1 — LDS: Low frequency down-sweep; DS: Down-sweep; DSS: Down-sweep series; LPS: Low frequency pulse series; LFPT: Low fre-quency fast pulse train; PS: Pulse series; FPT: Fast pulse train; DSS: Down-sweep series; RPS: Regular pulse series; APPPS: Pulse series with alternating pulse period; UFPS (kwa): Ultra-fast pulse series. (FFT: 8192; Hamming window, 50% overlap) [file peerj-09-12551-s001.pdf]

SUPPLEMENTARY MATERIAL 1.

Spectrograms of the 10 occurring sounds identified as fish calls. LDS: Low frequency down-sweep; DS: Down-sweep; DSS: Down-sweep series; LPS: Low frequency pulse series; LFPT: Low frequency fast pulse train; PS: Pulse series; FPT: Fast pulse train; DSS: Down-sweep series; RPS: Regular pulse series; APPPS: Pulse series with alternating pulse period; UFPS (kwa): Ultra-fast pulse series. (FFT: 8192; Hamming window, 50% overlap)

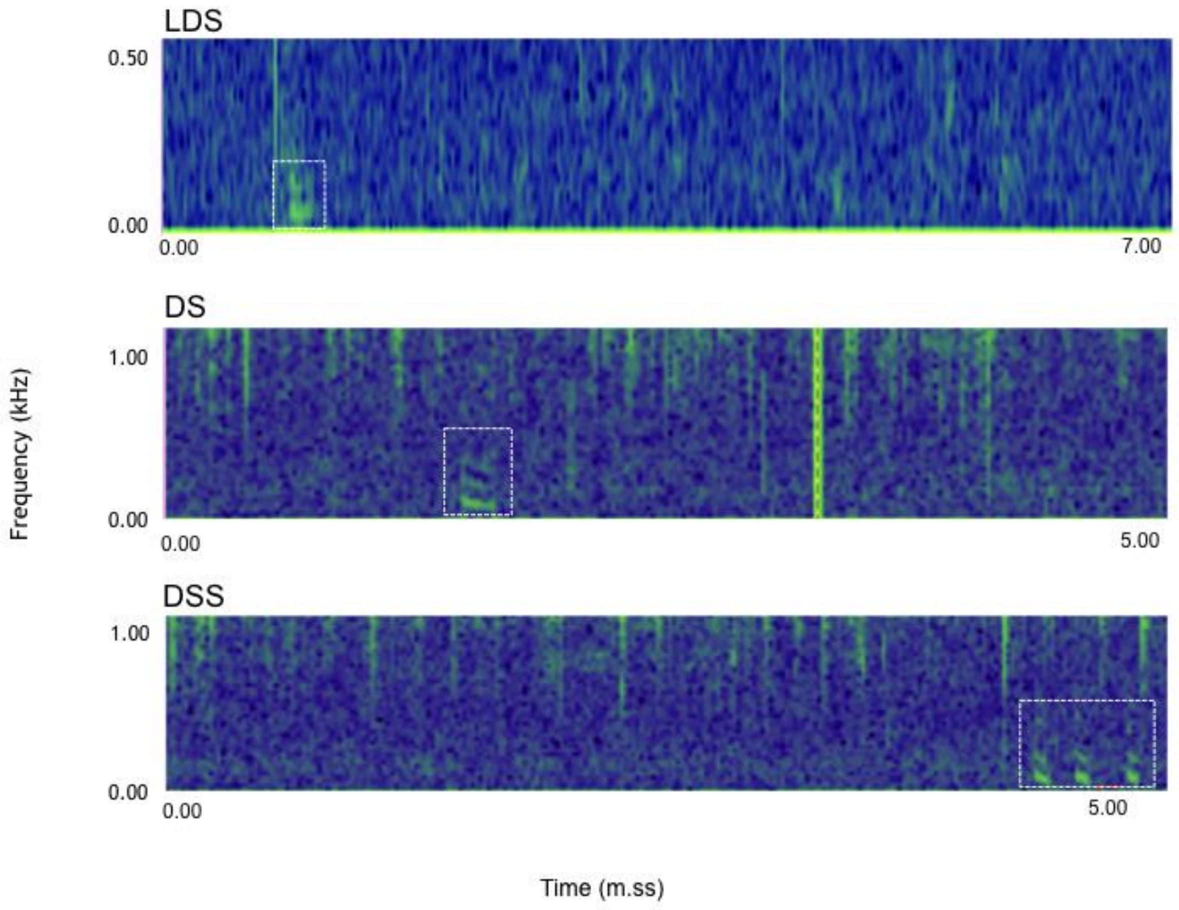

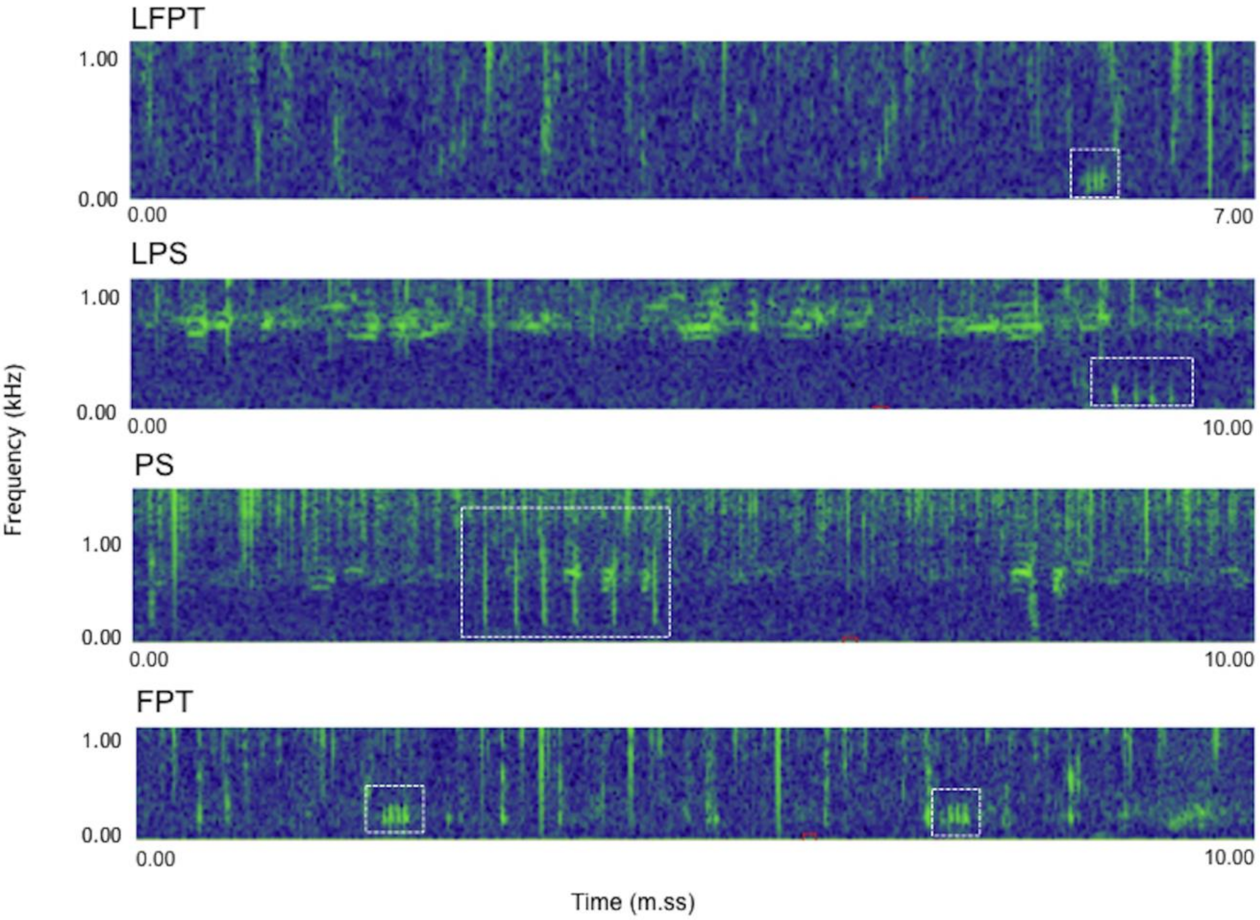
LFPT
1.00
0.00
0.00
7.00
LPS
1.00
0.00
0.00
10.00
PS
1.00
0.00
0.00
10.00
FPT
1.00
0.00
0.00
10.00
Frequency (kHz)
Time (m.ss)

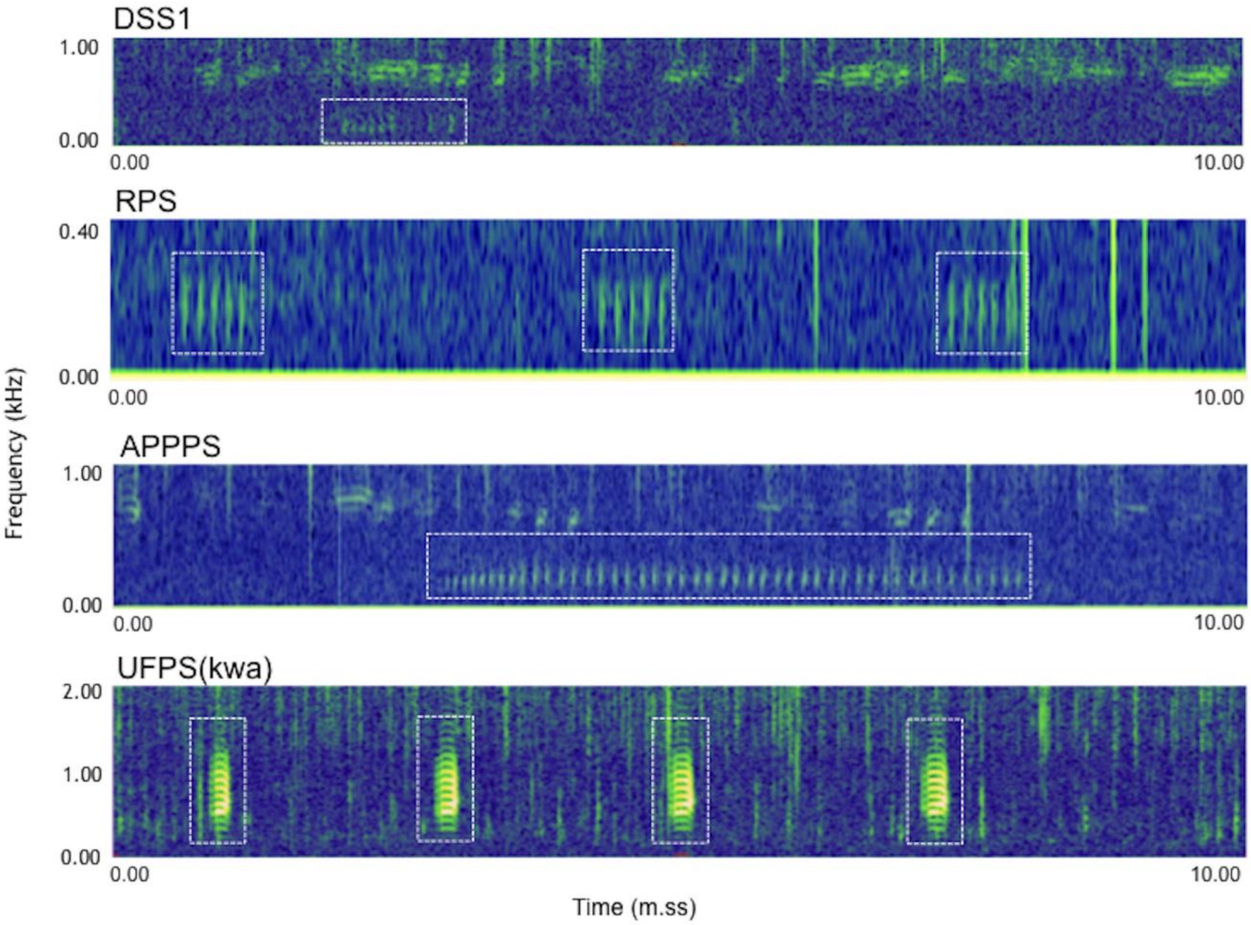
DSS1
1.00
0.00
0.00
10.00
RPS
0.40
0.00
0.00
10.00
APPPS
1.00
0.00
0.00
10.00
UFPS(kwa)
2.00
1.00
0.00
0.00
10.00
Frequency (kHz)
Time (m.ss)
