# Supplementary figures and images for: Marine soundscape and fish biophony of a Mediterranean marine protected area
Source: PeerJ. 2021 Dec 15;9:e12551. doi: 10.7717/peerj.12551 (PMC8684326; doi:10.7717/peerj.12551)

BR - 10 am

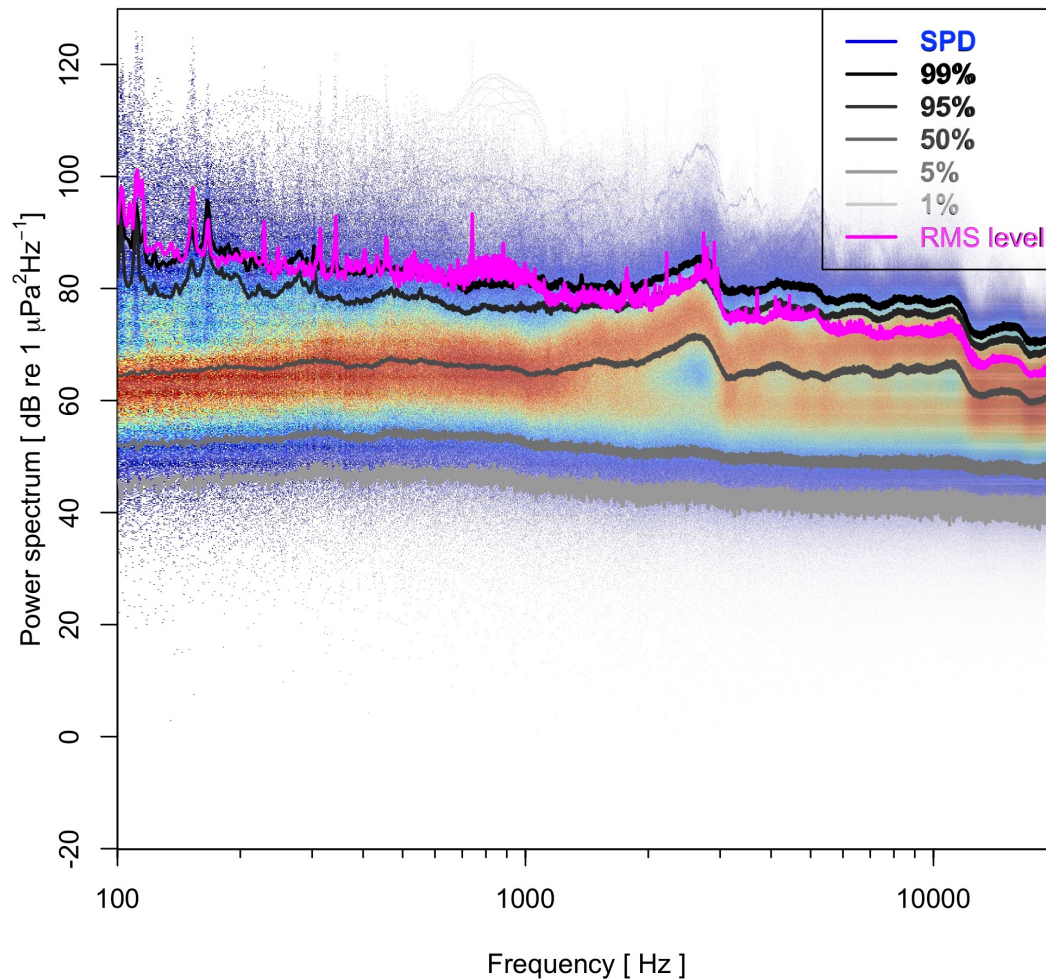

BR - 12 AM

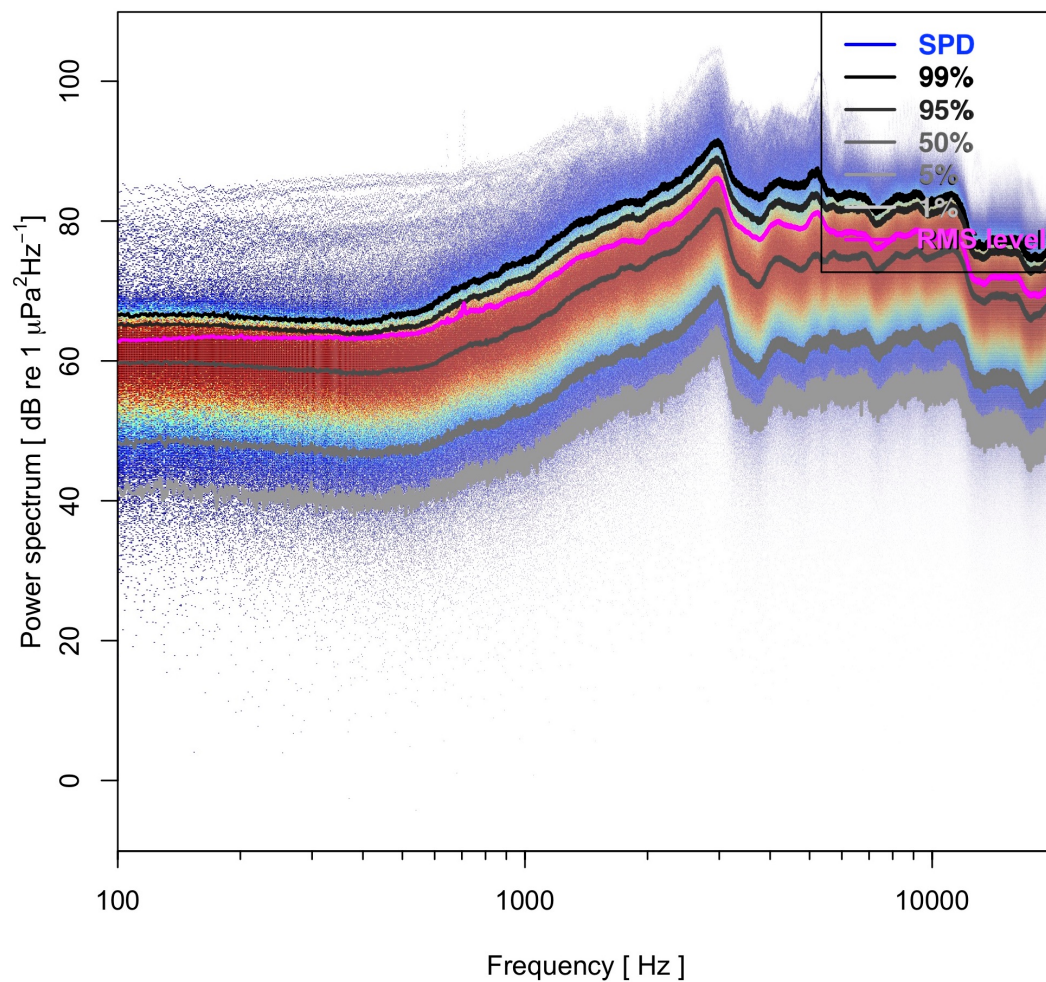

PG - 10 AM

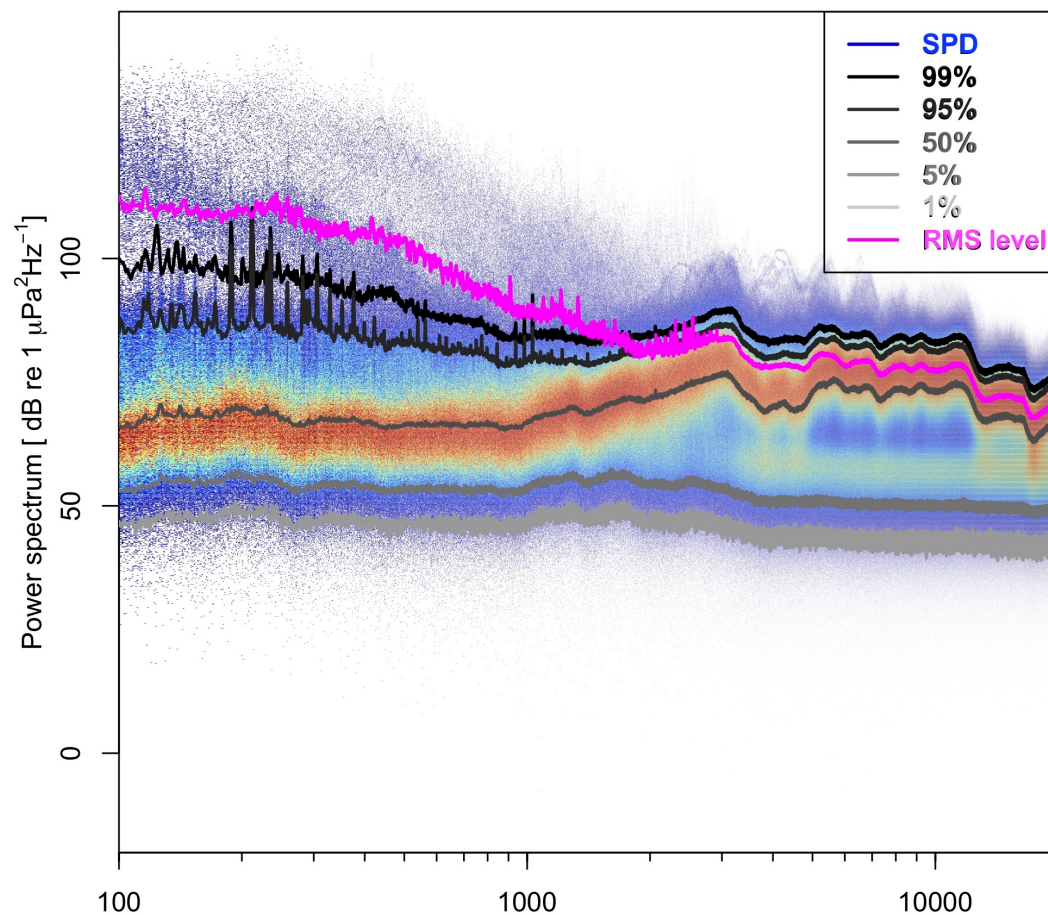

PG - 12 AM

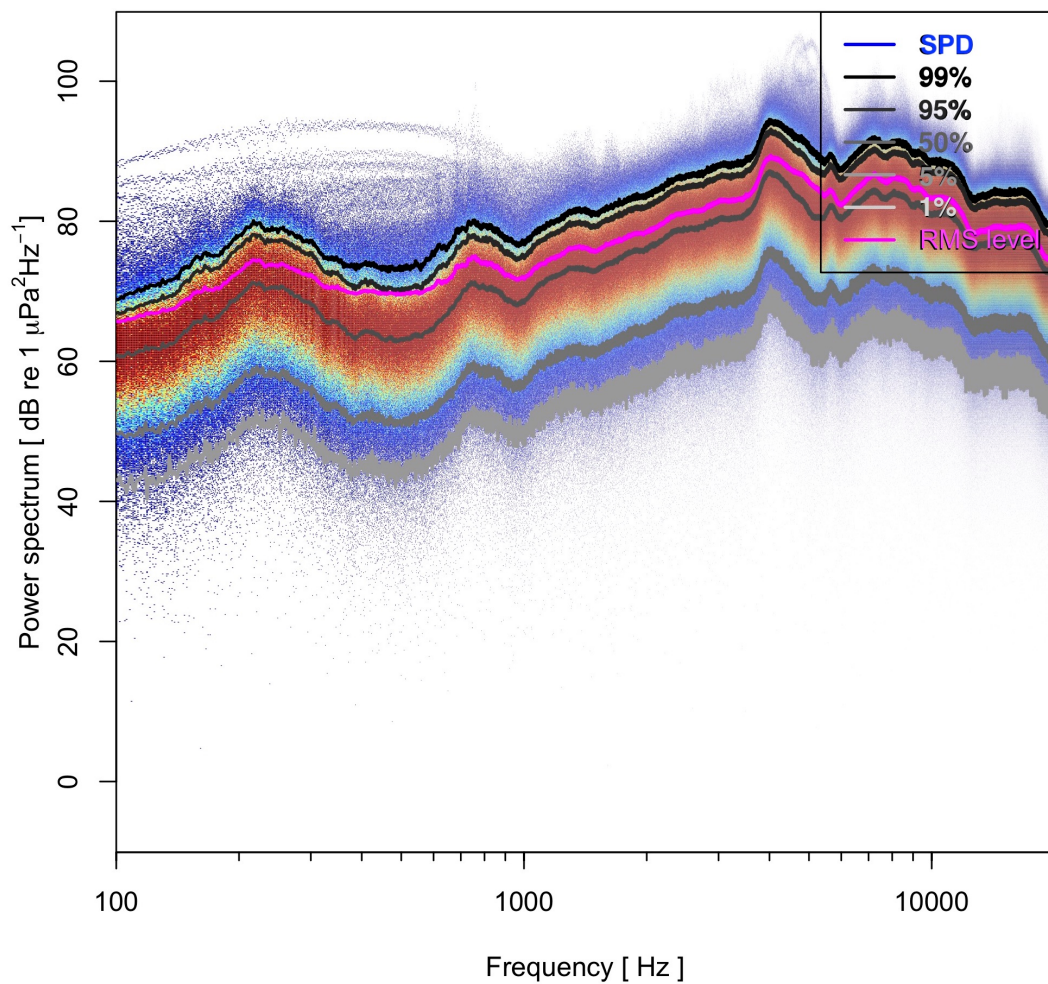

SA - 10 am

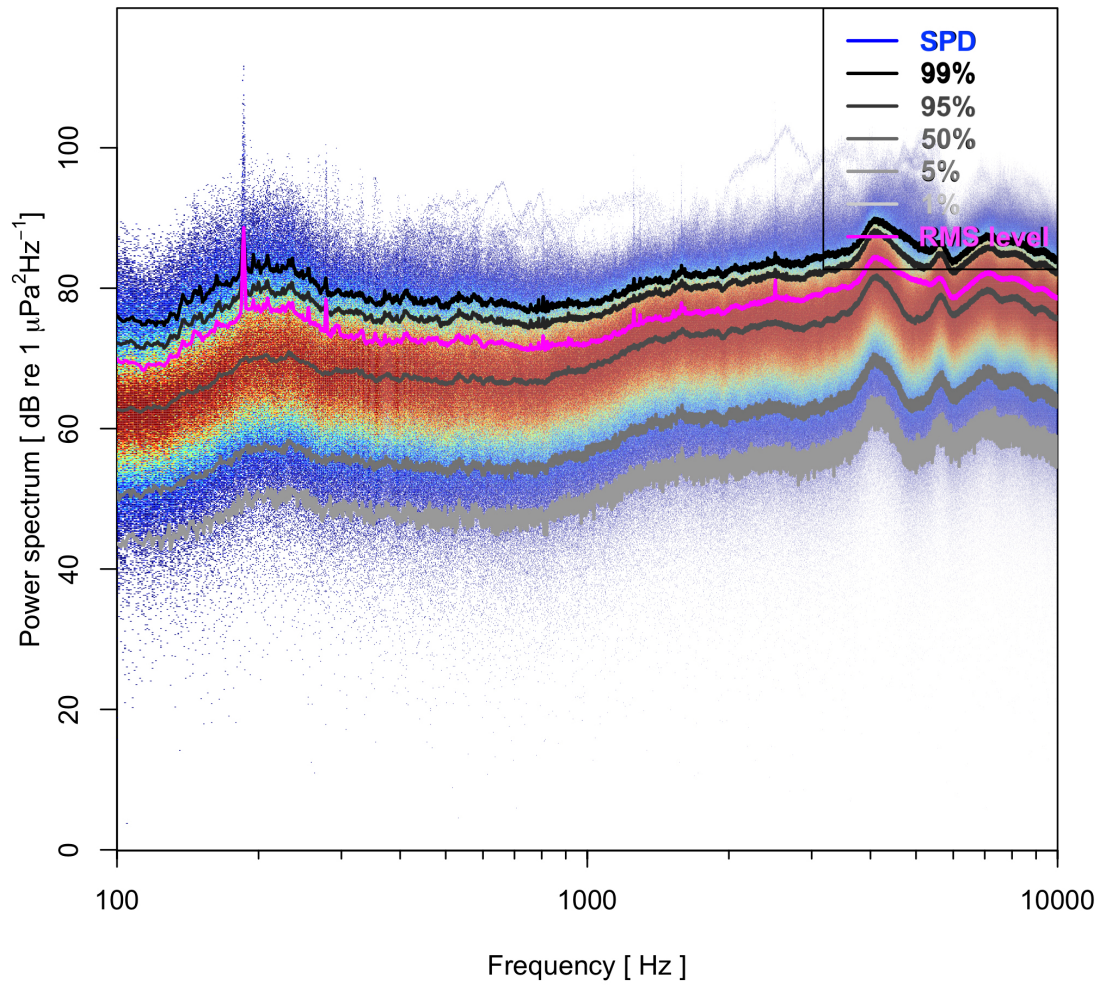

SA - 12 AM

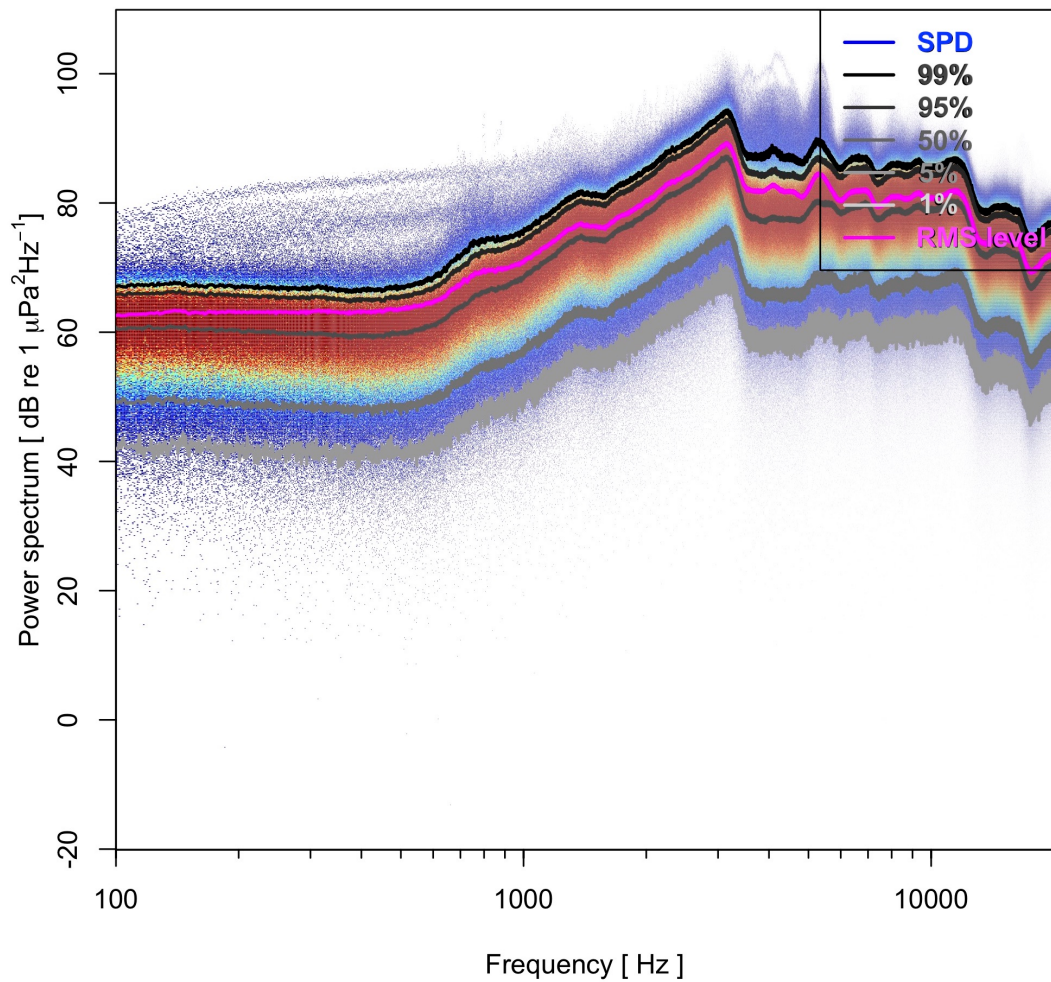

Supplement: Supplemental Information 2 — Example of spectral probability densities over 1 h, in the range 100 to 16,000 kHz, at 10 am and 12 am. BR: Bramassa; PG: Punta Giglio; SA: Sant’Antonio. [file peerj-09-12551-s002.pdf]
